# Three‐Dimensional Magnetic Bioprinting Spheroids as an In Vitro Model to Study the Oviductal Physiology

**DOI:** 10.1002/mrd.70049

**Published:** 2025-08-21

**Authors:** Patricia Kubo Fontes, Ana Beatriz Florencio da Silva, Ana Beatriz dos Reis Bartoli, Thays Antunes, Arnaldo Rodrigues dos Santos Júnior, Marcella Pecora Milazzotto

**Affiliations:** ^1^ Center of Natural and Human Sciences Federal University of ABC Santo André SP Brazil; ^2^ Center of Natural and Human Sciences Federal University of ABC São Bernardo do Campo SP Brazil

**Keywords:** bioprinting, cytokeratin, extracellular matrix, OVGP1, vimentin

## Abstract

In vitro models to study the oviduct are challenged by cellular dedifferentiation, a complex coculture system for embryo production, limited cell lifespan, and/or very complex methodologies. Hence, we aimed to develop an in vitro oviductal model using the magnetic bioprinting system, a three‐dimensional (3D) culture system. Using the bovine epithelial and stromal oviductal cells (BOEC and BOSC, respectively), we produced the Oviductal Magnetic Spheroid (OMS), a duo somatic cell spheroid aggregate with self‐organization capacity. The OMS showed to be viable for 21 days and recapitulated the oviductal tissue features after 7 days in culture, such as a simple epithelial cell layer facing outwards, expressing ciliation (acetylated tubulin positive) and secretory marker (oviduct‐specific glycoprotein 1). Although the responsiveness for hormonal treatment with estradiol and progesterone in an estrous cycle‐dependent way might require further improvements, the OMS offers an ethical and practical alternative as a three‐dimensional oviductal in vitro model to study oviductal physiology, and maybe, a future platform to test therapies and a technology aiming to improve fertility and assisted reproduction success.

## Graphical abstract

The oviductal magnetic spheroid (OMS) is a three‐dimensional in vitro model produced by the magnetic bioprinting system. Resembling the oviductal tissue, the OMS is formed by epithelial and stromal oviductal cells, that, after magnetization with nanoparticles and placed in a magnetic field, result in a cell aggregate.

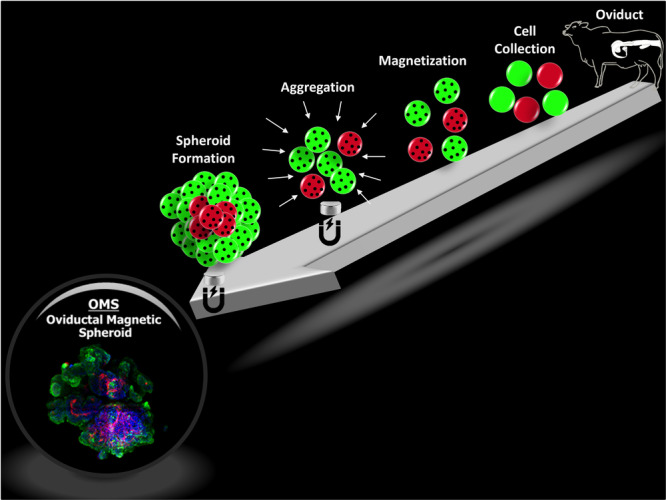

## 1 Introduction

Early embryonic mortality is one of the most critical problems affecting bovine reproduction, which can result in up to 40% reproductive failure (Humblot 2001). More specifically, up to 23% of embryo loss occurs before day four, when the embryo is still in the oviduct (Reese et al. 2020). As the organ responsible for providing the optimal microenvironment for gametes' final capacitation and maturation, fertilization process, and early embryo development, the study of the oviduct has been in the spotlight as a promising strategy to increase pregnancy success. However, the in situ access for the oviduct is challenging, and the lack of real‐time in vivo sampling and observations of the oviduct has become bottleneck in getting a deeper understanding of this organ's functionality. Moreover, the high costs and invasive surgical procedures (as an alternative to animal slaughter) limited the advancement of understanding oviductal physiology using animals (Humblot 2001; Ménézo and Hérubel 2002).

Accordingly, in vitro models offer an ethical and practical alternative to traditional animal models. Studying the oviduct cells under in vitro conditions has gathered great progress and knowledge using a monolayer (2D) culture system (Wijayagunawardane et al. 2005). However, a three‐dimensional (3D) culture system reproduces higher accuracy in the cell structure and its complex physiological mechanisms (Vinci et al. 2012). In this context, several 3D culture systems have been standardized for oviductal cells, such as organoids (Bourdon et al. 2021; Kessler et al. 2015; Menjivar et al. 2023), microporous inserts (Chen et al. 2018, 2013b; Ferraz et al. 2018; Palma‐Vera et al. 2014) (associated with microfluidics (Ferraz et al. 2018)), explants (Gualtieri et al. 2013; Lefebvre et al. 1997; Rottmayer et al. 2006; Walter 1995), and vesicle‐like structures (also called spontaneous spheroids) (Mahé et al. 2023; Pranomphon et al. 2024; Xu et al. 1992). Each 3D culture system has its specific characteristics, benefits, and limitations. In this regard, we noticed a weakness in the oviductal models, a system that supports the coculture of different cell types, which facilitates cell–cell and cell–matrix interactions.

Within this respect, spheroid aggregate cell culture is a promising 3D culture technique that allows multicellular tissue modeling. Several methods are available for cellular spheroids fabrication, such as microfluidics, spinner culture, agarose microwells, hanging drops, and magnetic fields (Cui et al. 2017; Lin and Chang 2008). Among them, the magnetic 3D (m3D) system has been described as producing an extracellular matrix (ECM) within 30 min of culture (Haisler et al. 2013; Tseng et al. 2013). This, combined with in vivo‐like cell–cell and cell–ECM interaction, has been associated with faster spheroid formation (Caleffi et al. 2021; Tseng et al. 2015). Based on the spatial variance of the magnetic field, the m3D system is divided into levitation (m3DL) and bioprinting (m3DB) systems and has been applied mainly to the tumor research field (Caleffi et al. 2021). Yet, it has been used in reproductive field studies as well (e.g., low‐grade ovarian carcinoma (Natânia de Souza‐Araújo et al. 2020), ovarian follicular growth (Antonino et al. 2019), and uterine myometrial cells (Souza et al. 2017)). However, to our best knowledge, its application with healthy oviductal cells aiming at the study of this tissue physiology is still missing.

Hence, our first hypothesis was that a spheroid culture system might provide an in vitro 3D model to study the oviductal tissue physiology. By providing a system that supports the epithelial and stromal cell coculture, our second hypothesis was that this model would present a higher accuracy on the complexity of the oviductal tissue organization. To test our hypothesis, we aimed to standardize an oviductal spheroid model using the m3DB system.

## 2 Results and Discussion

**Figure 1:**
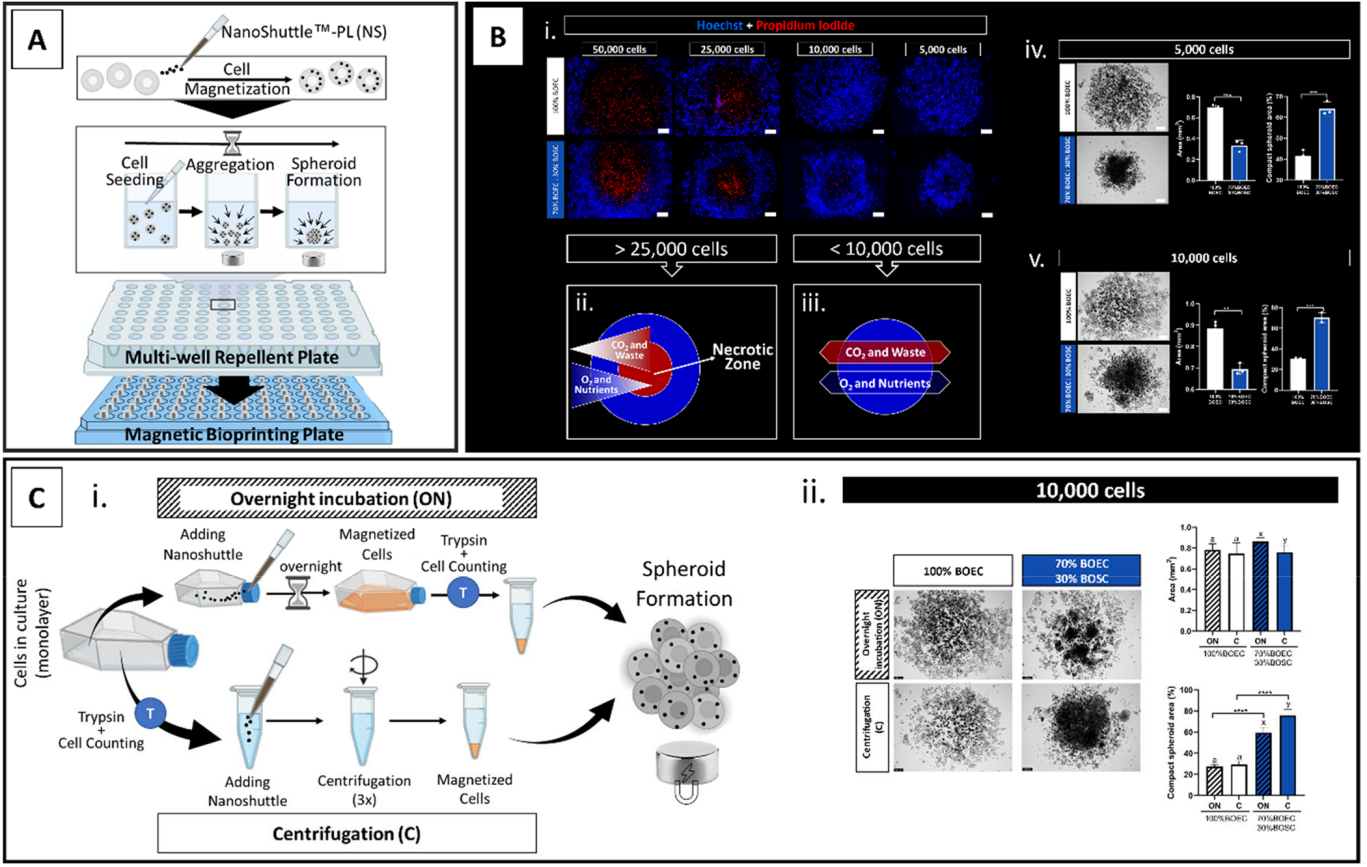
Elementary standardization steps for spheroid formation: spheroid cell number size and cellular magnetization methods. (A) Schematic representation of magnetic 3D Bioprinting (m3DB) system; (B) Spheroid cell number size evaluation (Experiment I) at 48 h after cell seeding: (i) 50,000, 25,000, 10,000, and 5000 cells per well formed by BOEC‐only (100% BOEC, white bars) or BOEC/BOSC‐mix (70/30%, blue bars) stained with Hoechst 33342 (nuclei) and Propidium Iodate (PI, necrotic‐positive cell marker); (ii) schematic representation of the necrotic zone formation in big spheroids ( > 25,000 cells); (iii) schematic representation of absence of necrotic zone formation in small spheroids ( < 10,000 cells); (iv) 5000 cells spheroids (representative images, bright‐field), and the graphical results of spheroid area (square millimeter—mm^2^) and spheroid compaction level (percentage, %), (v) 10,000 cells spheroids (representative images, bright‐field), and the graphical results of spheroid area (mm^2^) and spheroid compaction level (%), ** and *** indicates *p* < 0.01 and 0.001, respectively; (C) Cellular magnetization methods evaluation (Experiment II) at 48 h after cell seeding: (i) schematic representation of cell magnetization by overnight incubation (ON) or centrifugation (C), starting with the cell in monolayer culture, followed by trypsinization step (T) before being seeded in the 96‐well non‐adherent plate and incubated with the Magnetic Bioprinting Plate; (ii) 10,000‐cell spheroids: representative images (bright‐field) of BOEC‐only (100%BOEC) or BOEC/BOSC‐mix (70/30%) and the graphical results of spheroid area (mm^2^) and spheroid compaction level (%), different letters represent statistical differences (*p* < 0.05) among magnetization methods in BOEC‐only (a‐b) or BOEC/BOSC‐mix (x‐y), **** represents statistical differences (*p* < 0.0001) when comparing BOEC‐only vs. BOEC/BOSC‐mix spheroids under the same magnetization methods. All data are presented as mean ± standard deviation (SD) with single spheroid values represented in the graphs as a circle. All scale bars represent 100 μm. Abbreviations: BOEC, bovine oviduct epithelial cells; BOSC, bovine oviduct stromal cells.

The magnetic 3D bioprinting (m3DB) cell culture method relies on magnetizing cells with nanoparticles, followed by applying a magnetic field to attract cells to form a spheroid at the bottom of a multi‐well repellent plate (Figure 1A). Developed by Souza et al. 2010), the NanoShuttle‐PL (NS) is a nanoparticle assembly smaller than 50 nm in size consisting of gold, iron oxide, and poly‐l‐lysine. By electrostatically attaching to the cell membrane, the NS is responsible for cell magnetization. Notably, these particles have been demonstrated to be nontoxic, do not affect cell proliferation and metabolism, and do not induce pro‐inflammatory responses and oxidative stress (Caleffi et al. 2021; Souza et al. 2010).

The first goal of this study was to evaluate the viability and applicability of using frozen oviductal cells for cell culture. By using frozen cells, the inconvenience and higher variability of multiple oviductal collections at a slaughterhouse would be reduced. As a result, freshly collected and frozen storage bovine oviductal epithelial cells (BOEC) and stromal cells (BOSC) were similar in terms of cell growth capacity and morphology features (Figure S1). Based on this, the whole study was performed using a cell bank collected all at the same time. In this experiment, we also confirmed the BOEC and BOSC purity using specific epithelial and stromal cell markers (anti‐cytokeratin and anti‐vimentin, respectively, Figure S1). Both cell types were collected exclusively from the ampulla segment of the oviduct. It is relevant that each segment is studied separately to avoid experimental bias and misleading because each oviductal segment shows a specific transcriptional, morphological, and anatomical profile consistent with its functionality (Gonella‐Diaza et al. 2015, 2017; Ito et al. 2016; Mahé et al. 2022; Yániz et al. 2000). Consequently, we selected the ampulla since it is the greatest oviductal fluid producer (Kavanaugh et al. 1992; Mahé et al. 2022), it is involved with the maturation of the gametes (Kölle et al. 2009), and it is the site of fertilization and early embryo development in cattle (Kölle et al. 2009).

Hence, the following sections describe the steps for the oviductal spheroid standardization using the m3DB system, mainly focusing on a stable structure that would resist pipette manipulation and culture media changes, have a relatively long‐term culture viability, and correspond to oviductal tissue features, aiming at its application as an in vitro oviductal model. Specifically, in Experiments I–IV, we present the steps to obtain a stable spheroid structure, followed by long‐term culture viability (Experiment V), and finally, the oviductal tissue features and hormone responsiveness (Experiments VI–VIII).

### 2.1 Experiment I: Impact of the Cell Number Size Per Spheroid

The m3D system is a one‐to‐one system: one well, one spheroid. Specifically for a 96‐well plate, spheroids from 1500 to 50,000 cells per well have been optimized in other tissue/organ conditions (Haisler et al. 2013). Therefore, in Experiment I, we evaluated the cell number parameter for oviductal cells, testing 50,000, 25,000, 10,000, and 5000 cells per well, either using BOEC‐only (100%BOEC) or BOEC/BOSC‐mix (70%/30%, respectively). After 48 h on the Magnetic Bioprinting Plate (Mag‐plate), spheroid viability was evaluated using Propidium Iodide (PI) dye as a cell necrotic marker. As a result, the 50,000 and 25,000‐cell spheroids (either BOEC‐only or BOEC/BOSC‐mix) presented a necrotic center as indicated by the high number of PI‐positive cells in the central spheroid area (Figure 1B‐i). This necrotic region is a well‐known component observed in spheroid systems, mainly caused by a lack of nutrition and oxygen in the interior zone (Figure 1B‐ii) (Yan et al. 2021). Although this feature is desired when studying solid tumors (mimicking the in vivo tumor conditions zones: the proliferative (external), senescence (medial), and necrotic zone (internal) (Tredan et al. 2007)), it does not support our goal to obtain an in vitro physiological oviductal model. Thus, the presence of a necrotic center was determined as an exclusion criterion. Consistent, the 5000‐ and 10,000‐cell spheroids (both BOEC‐only and BOEC/BOSC‐mix) were necrotic center‐free (Figure 1B‐i/iii), indicating great cell viability. Although it was viable, cells appeared to be scattered in the well, and in a more evident way in the BOEC‐only than in the BOEC/BOSC‐mix spheroids (Figure 1B‐iv/v). Therefore, two measurements were accessed as an indicator factor of cell dispersion: (i) the spheroid area and (ii) the spheroid compactness level. The spheroid area was related to that; the bigger the area, the more scattered the cells are (with the opposite also true). The spheroid compactness level was related to the fact that the higher the compactness level, the less scattered the cells are (with the opposite also true). As a result, the BOEC/BOSC‐mix spheroids presented a smaller area compared to BOEC‐only spheroids, either in the 5000‐cell (Figure 1B‐iv) and the 10,000‐cell spheroids (Figure 1B‐v), which was indeed reversely related to the spheroid compaction level (Figure 1B‐iv/v). Although these parameters suggested that the BOEC/BOSC‐mix spheroids were the most stable structure in this experiment, still the cells could not keep a singular structure once disturbed with a pipette. From Experiment I, we conclude that 5000‐ and 10,000‐cell spheroids are viable for culture, since they do not present a necrotic central region. Still, a higher compaction level is required to stabilize the oviductal spheroid. From this point, the 10,000‐cell size spheroids were adopted for all the following experiments. The 10,000‐cell spheroid was selected over the 5000‐cell spheroid because a structure with a higher cell number might increase the accuracy and reliability of future qualitative and quantitative experimental outcomes.

### 2.2 Experiment II: Evaluation of Different Cellular Magnetization Methods

Since the previously accomplished spheroids' compaction level (Experiment I) was not enough to stabilize the oviductal spheroid, in Experiment II, another cell magnetization method was applied in an attempt to improve this feature. Cells magnetized by centrifugation (C), as applied in Experiment I, were compared to cells magnetized by overnight incubation (ON). For the overnight incubation, the NS particles were kept with the cells still in the monolayer culture for at least 18 h (Figures 1C‐i and S2A), meaning that the trypsinization and cell counting steps are performed afterward. On the other hand, for the magnetization using the centrifugation procedure, cells are trypsinized and counted before incubation with the NS particles, allowing the magnetization procedure of a desired number of cells (Figures 1C‐i and S2A). Following the manufacturer's instructions, the benchmark concentration is 200 μL NS/T‐25 flask and 1 μL NS/10,000 cells for magnetization by overnight incubation and centrifugation methods, respectively. Therefore, the magnetization by the centrifugation method has the advantage over the overnight incubation by not magnetizing cells above the required amount, consequently, saving costs of the NS particles. On the contrary, the magnetization by overnight incubation submits the cells to less cellular mechanical stress, saving cells from three extra rounds of centrifugation, which can be survival‐determinant for some cell types and lines.

To test the magnetization methods in Experiment II, cells were seeded at 10,000 cells per well, either BOEC‐only (100%BOEC) or BOEC/BOSC‐mix (70%/30%), and incubated in the Mag‐plate (48 h). As a result (before spheroid formation), BOEC could not be magnetized by the overnight method (noticed by NS clumps floating in the culture media, Figure S2B, white arrows), but it worked in the centrifugation method (noticed by brownish cell pellet color, Figure S2B). The BOSC could be magnetized by both methods: noticed by the cell monolayer's peppered‐like appearance after the overnight incubation method, and the brownish cell pellet color after three centrifugation rounds (Figure S2C). Regarding the spheroid compactness level, the BOEC‐only spheroids were significantly less compact than the BOEC/BOSC‐mix spheroids (independently of the magnetization method and spheroid cell number, Figure 1C‐ii). The centrifugation method was more efficient in providing a lower area and higher compaction level than the overnight incubation method in BOEC/BOSC‐mix spheroids (Figure 1C‐ii). Although the BOEC/BOSC‐mix spheroids derived from the centrifugation method were the most compact, once again, the cells could not keep a singular structure after being disturbed with a pipette. As conclusions of Experiment II, we observed that: (i) cell magnetization using the centrifugation method is better for both BOEC and BOSC, (ii) BOEC/BOSC‐mix spheroids are indeed more compact them BOEC‐only, and (iii) a higher compaction level is required to stabilize the oviductal spheroid. From this point, the magnetization by centrifugation was adopted for all the following experiments.

### 2.3 Experiment III: Evaluation of Cell Seeding Protocols and Cell Proportion (BOEC/BOSC)

**Figure 2:**
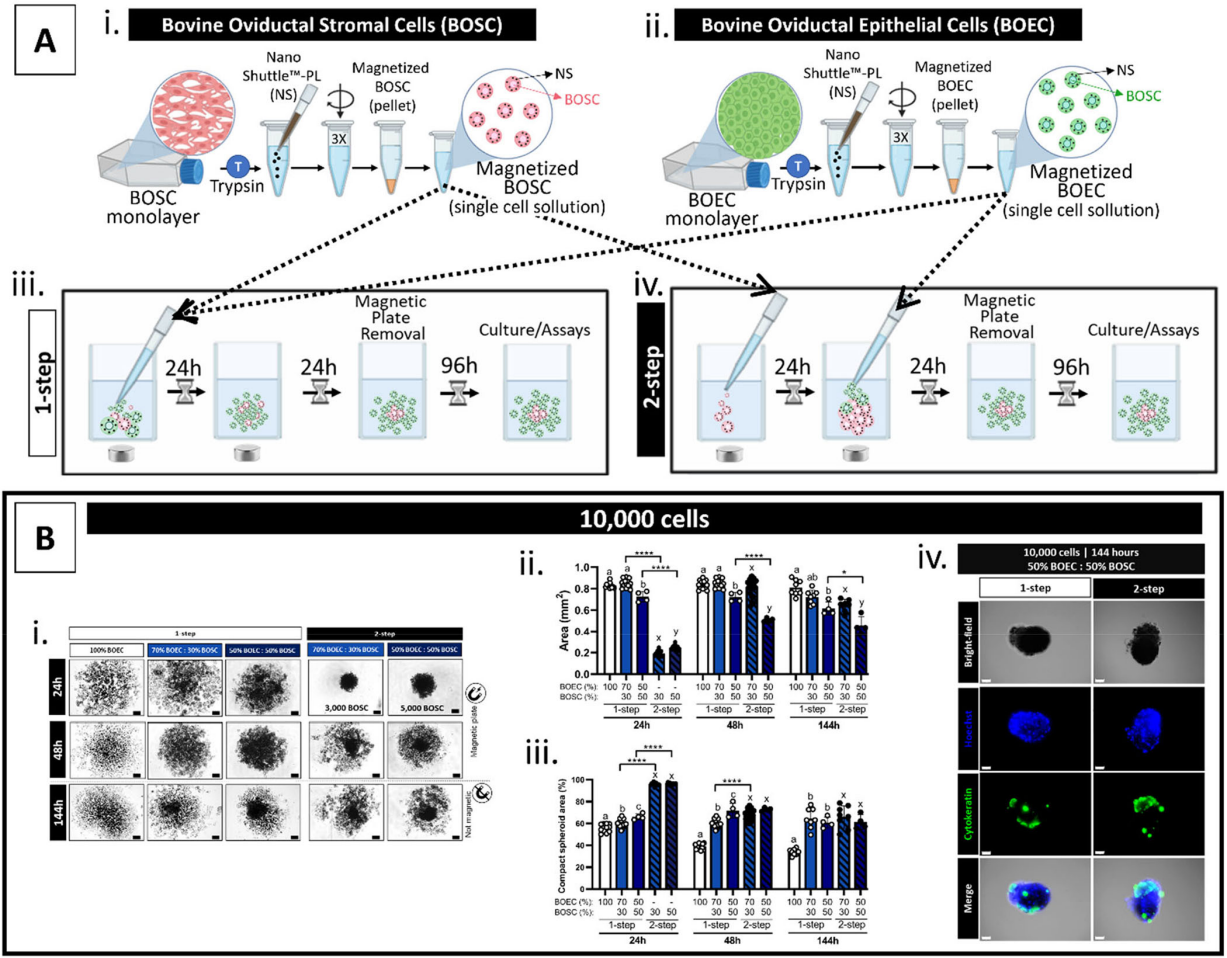
Improvement of spheroid formation: cell seeding protocols and cell proportion (BOEC/BOSC). (A) Schematic representation of one‐ or two‐step cell seeding protocols, showing the cell seeding order and the duration of each step, (B) results of experiment III of 10,000‐cell spheroids evaluated at 24, 48, and 144 h after cell seeding (i) representative images (bright‐field) of the spheroids formed with BOEC‐only (100%BOEC, white bars) and BOEC/BOSC‐mix (70/30%, lighter blue bars, and 50/50%, darker blue bars) at three time‐points and the graphical results of (ii) spheroid area (mm^2^), (iii) spheroid compaction level (%), and (iv) immunostaining in 144 h spheroids for epithelial cell marker (anti‐cytokeratin, green color) and Hoechst 33342 (DNA, blue); different letters represent statistical differences (*p* < 0.05) among different cell proportion in one‐step (a‐c) or two‐step (x‐y) protocols, * (*p* < 0.05) and **** (*p* < 0.0001) represents statistical differences when comparing cell seeding protocols (one‐ vs. two‐step). All data are presented as mean ± standard deviation (SD) with single spheroid values represented in the graphs as a circle. All scale bars represent 100 μm. Abbreviations: BOEC, bovine oviduct epithelial cells; BOSC, bovine oviduct stromal cells.

Based on the fact that the BOEC/BOSC‐mix spheroids have shown better compaction levels than the BOEC‐only (Figure 1B, C), in Experiment III, the BOSC was further explored, associating two strategies: (i) cell seeding protocol and (ii) cell proportion. After magnetized by centrifugation (Figure 2A‐i/ii), BOSC and BOEC were seeded simultaneous in the wells (defined as a one‐step protocol, Figure 2A‐iii), or the BOSC was seeded 24 h before the BOEC seeding (defined as a two‐step protocol, Figure 2A‐iv). For cell proportion, BOEC and BOSC were mixed as 100%/0% (i.e., BOEC‐only), 70%/30%, and 50%/50%, respectively. To assess the impact of higher BOSC proportion and/or its role as a support structure for the spheroid formation, spheroid features were analyzed at 24, 48, and 144 h after the first cell seeding (Mag‐Plate was removed at 48 h). Corroborating the previous results, bigger areas' spheroids had lower compaction levels (Figure 2B‐i/ii/iii). At 48 h, the spheroid compaction level was affected by the cell seeding protocol (higher in the two‐step than the one‐step protocol, Figure 2B‐iii) and by the cell proportion (highest at 50/50 compared to 70/30 and 100/0, Figure 2B‐iii). Curiously, at 144 h, except for BOEC‐only, all groups had similar spheroid compaction levels (independent of BOSC proportion and cell seeding protocol, Figure 2B‐iii) and presented a central darker area under the bright‐field microscope (Figure 2B‐i). At 48 h, this central darker area was only noticeable in the two‐step conditions (Figure 2B‐i), which seems very similar to the BOSC‐only structures at 24 h (two‐step). These 24 h BOSC‐only structures had a high compact level (above 95%, either in the 70/30 or 50/50 conditions, Figure 2B‐i/iii). Consequently, two hypotheses were raised: (i) this darker area consists of BOSC‐only, meaning that the BOEC could not be incorporated into the spheroid body; or (ii) BOEC has been seeded above an ideal quantitative limit to be incorporated into the spheroid body. Therefore, the presence of epithelial cells was evaluated on these central darker structures (one‐ and two‐step, from 50/50 condition, at 144 h) by immunostaining (anti‐cytokeratin), revealing a low number of BOEC, restricted to a few spots (green marker, Figure 2B‐iv). Interestingly, they kept a singular structure after being disturbed with a pipette. Collectively, from Experiment III, we conclude that: (i) BOSC are necessary for spheroid formation, (ii) seeding BOSC first in a higher proportion accelerates the delivery of a central compact structure, however, later these factors become non‐essential, and (iii) low number of BOEC are being incorporated in the central compact structure.

In general, the spheroid formation involves three critical steps: (i) dispersed cells must first get closer to forming loose aggregates (which occurs by the gentle magnetic force in the m3DB system); (ii) this cell–cell contact triggers the second step, consisting of cadherin expression upregulation; (iii) leading to the third step, a more compact form is obtained due to the homophilic cadherin‐cadherin binding and cell–ECM interaction (reviewed by (Cui et al. 2017)). Because different cell types have different adhesion molecule profiles and secrete ECM at different rates (within a variety of content and content proportions), the efficiency in forming spheroids is different (Lin et al. 2006; Robinson et al. 2004). Moreover, pathological cellular status has also been related to affect cell adhesion. For instance, epithelial cell adhesion molecule (EpCAM), a transmembrane glycoprotein involved in cell‐cell adhesion, is overexpressed in various epithelial cancers (Goossens‐Beumer et al. 2014). When considering this information, stromal cells, in general, are more likely to become a compact structure due to their cell–cell adhesion molecules and cell–ECM interaction capacity (Cui et al. 2017). In our study, even though ECM production and cell adhesion markers were not assessed, the formation of a stable BOSC‐only spheroid is indicative that these stromal cells can interact with each other, while the BOEC cannot. Based on that, we hypothesized that the ECM could be the missing factor in providing anchorage and mechanical support for the BOEC adhesion. Hence, the effect of adding commercial ECM content was evaluated in Experiment IV.

### 2.4 Experiment IV: Evaluation of Culture Media Supplementation With Extracellular Matrix (ECM)

**Figure 3:**
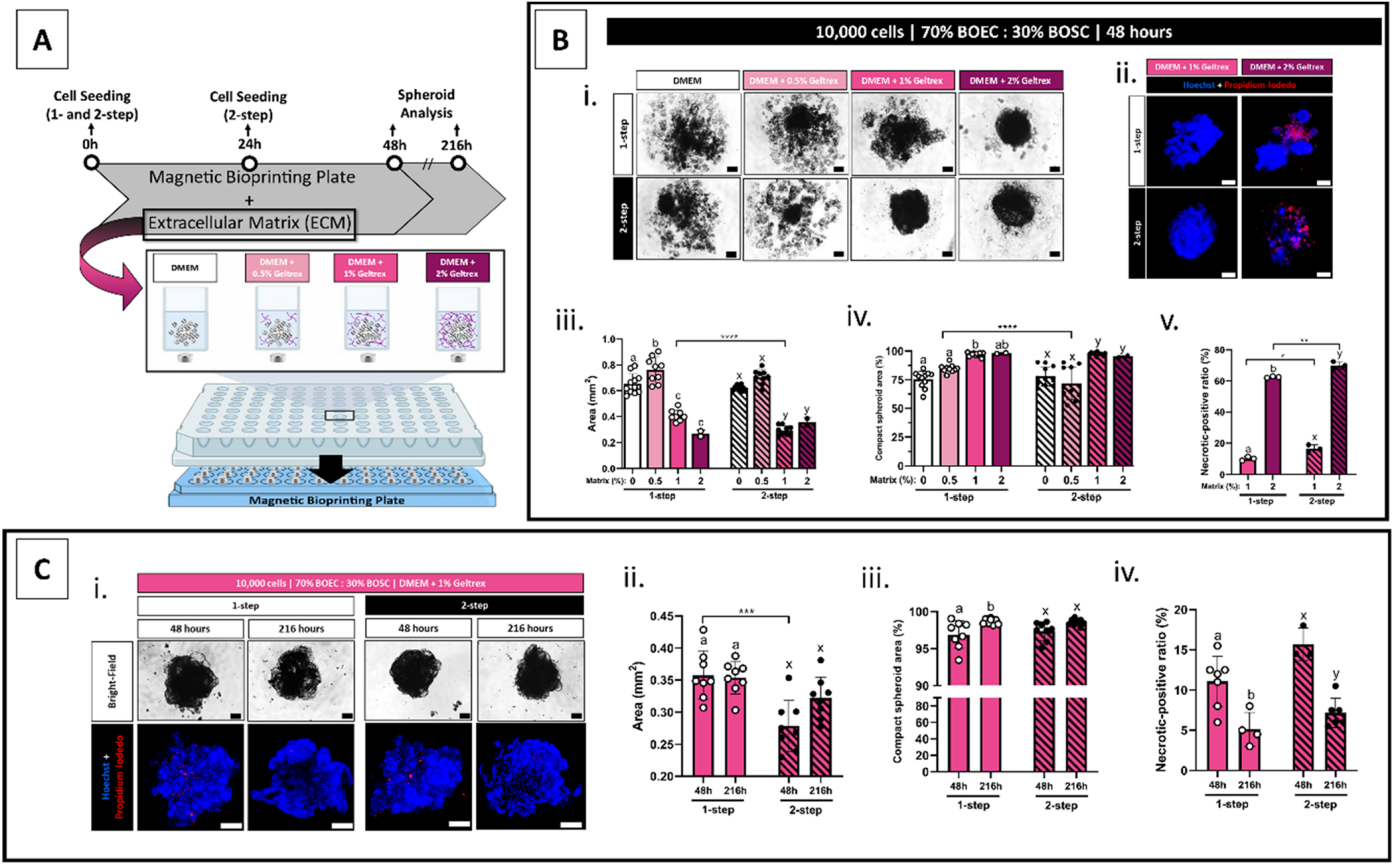
Spheroid stability with culture media supplementation with extracellular matrix (ECM). (A) schematic representation of the experimental design, 10,000‐cell spheroids consisting of BOEC/BOSC‐mix (70/30%) were seeded as one‐ or two‐step protocol with culture media supplemented with 0, 0.5, 1, or 2% v/v Geltrex supplementation, cells were incubated in the supplemented media and on top of the Magnetic Bioprinting Plate for 48 h, following the culture without them until 216 h in total. (B) Analysis at 48 h of culture: (i) representative images (bright‐field), (ii) representative images of spheroids stained with Hoechst 33342 (nuclei) and Propidium Iodate (PI, necrotic‐positive cell marker), graphical results of (iii) spheroids area (square millimeter, mm^2^), (iv) spheroid compaction level (percentage, %), and (v) necrotic‐positive cell ratio (%); different letters represent statistical differences (*p* < 0.05) among different ECM supplementation groups in one‐step (a‐c) and two‐step (x‐y) protocols and * *p* < 0.05, ** *p* < 0.01, and **** *p* < 0.0001 represent statistical differences when comparing one‐ vs. two‐step protocols. (C) Analysis of spheroids cultured with 1% v/v Geltrex supplementation at 48 and 216 h of culture: (i) representative images of spheroids (bright‐field and Hoechst + PI), graphical results of (ii) spheroids area (mm^2^), (iii) spheroid compaction level (%), and (iv) necrotic‐positive cell ratio (%); different letters represent statistical differences (*p* < 0.05) among different culture time in one‐step (a‐b) and two‐step (x‐y) protocols and *** represents statistical difference (*p* < 0.001) when comparing one‐ vs. two‐step protocols. All data are presented as mean ± standard deviation (SD) with single spheroid values represented in the graphs as a circle. All scale bars represent 100 μm. Abbreviations: BOEC, bovine oviduct epithelial cells; BOSC, bovine oviduct stromal cells; NS, NanoShuttle‐PL.

In experiment IV, commercial ECM (Geltrex) was added to the culture media in different concentrations (0, 0.5, 1, and 2% v/v, Figure 3A). All these ECM supplementation concentrations were insufficient to solidify the culture media (kept in a liquid state). Spheroids were produced using the one‐ and two‐step seeding, testing 70%/30% (Figure 3) and 50%/50% (Figure S3) BOEC/BOSC proportions, and evaluated 48 h after seeding the first cells (Mag‐Plate removal time). As a result, the ECM supplementation endorsed the oviductal cell aggregation into a spheroid (Figures 3 and S3). In the spheroids from the 70%/30% cell proportion condition, the area was smaller in 1% and 2% compared to 0% and 0.5% groups (either in the one‐ or two‐step protocol, Figure 3B‐iii), which are inversely correlated to compactness, with compaction level higher in 1% and 2% compared to 0% and 0.5% groups (Figure 3B‐iv). Similar profiles of area and compaction level were observed in the spheroids from the 50%/50% cell proportion condition (Figure S3). The compaction levels above 95% in the 1% and 2% groups were high enough to keep the cells as a singular structure once it was disturbed with a pipette (either from one‐ or two‐step protocols and 70%/30% or 50%/50% groups). Interestingly, it was noticed in the 1% and 2% groups, the majority of the cells were incorporated into the spheroid structure, leaving almost no cells loose in the wells. However, in the 2% group, more than 50% of the cells were PI‐positive, and higher when compared to the 1% group (either in the one‐ or two‐step protocols, Figure 3B‐ii/v). Such necrotic cell ratios would be detrimental to the spheroid applicability; thus, the 2% group was ineligible for further evaluation.

Exploring the 1% v/v ECM group, the spheroid progression and behavior were evaluated over a longer period in culture. At the time of the Mag‐Plate removal (at 48 h), the culture media were completely replaced by culture media ECM‐free, and the spheroids were cultured for seven more days (216 h in total, including culture media changes every other day, Figure 2C). As a result, the spheroid area was smaller in the two‐step compared to the one‐step protocol at 48 h of culture (Figure 3C‐i/ii). Spheroids from the one‐step protocol presented a delay in achieving the highest compaction levels (lower compaction level at 48 h than 216 h), while the compaction in spheroids from the two‐step protocol was already at the highest level at 48 h (Figure 3C‐i/iii). Interestingly, the PI‐positive cell ratios were lower at 216 h compared to 48 h (either in one‐ and two‐step protocols, Figure 3C‐i/iv). The higher ratio observed at 48 h might be related to cells that die during the spheroid cell formation (which includes trypsin treatment and several centrifugation steps), whereas the dead cells might detach from the spheroid content throughout the culture days and media exchanges.

Because the ECM supplementation efficiently promoted a BOEC/BOSC‐mix spheroid formation, we also evaluated the ECM supplementation efficiency in BOEC‐only spheroids (10,000 BOEC, DMEM + 1% v/v Geltrex). Although the BOEC stayed in the center of the well while under the magnetic field (at 48 h), cells dispersed after Mag‐Plate removal without spheroid formation (at 216 h, Figure S4A). Contrary to BOSC‐only, which forms a compact spheroid in ECM‐free media (Figure S4A). Thus, from Experiment IV, we conclude that ECM supplementation is required for oviductal cell spheroid formation, but such supplementation has an optimal concentration to be applied. Moreover, this culture system supports different BOEC/BOSC‐mix conditions. Here, we demonstrated an equal success of 70%/30% and 50%/50% proportions in forming a compact spheroid (in the following experiments, only the 70%/30% proportion was evaluated).

Henceforth, the spheroid formed by epithelial and stromal primary oviductal cells was titled Oviductal Magnetic Spheroid (OMS). We also determined from now on that day zero was the coordinated day of the Mag‐Plate removal, simultaneous to the first culture media change (culture media replacement from 1% v/v Geltrex to an ECM‐free media). Although at first, the 48‐h incubation seemed enough for the spheroid formation, a 72‐h incubation (in the Mag‐Plate with the ECM‐supplemented media) improved spheroid stability without affecting the PI‐positive cell ratio when compared to 48 h (data not shown). Longer spheroid formation periods were not evaluated as they would probably impair cell survival and delay treatment evaluations. Nonetheless, the efficiency of the OMS formation protocol was verified by applying the two‐step protocol and cell incubation for 72 h in the Mag‐Plate and 1% v/v Geltrex media supplementation. A full OMS formation efficiency was demonstrated (96/96 OMS formed with BOEC from four different animals, Figure S4B), confirming the reproducibility of the protocol. In addition, the m3DB system showed high repeatability regarding the OMS diameter, with less than 13% of coefficient of variation (Figure S4C). Consequently, this final OMS formation protocol (Figure S5i‐iv) accomplishes advantages for this 3D oviductal model by allowing the building of a manageable, but highly reproducible and repeatable, spheroid structure (Figure S5v). Such features dismiss the need to select spheroids by size (Mahé et al. 2023; Pranomphon et al. 2024), allow working with spheroids of a desired cell number size (10,000–5000 per spheroid), and with different proportions of BOEC: BOSC (70%/30% and 50%/50%, respectively).

### 2.5 Experiment V: Evaluation of Long‐Term Culture of Oviductal Magnetic Spheroids (OMS)

**Figure 4:**
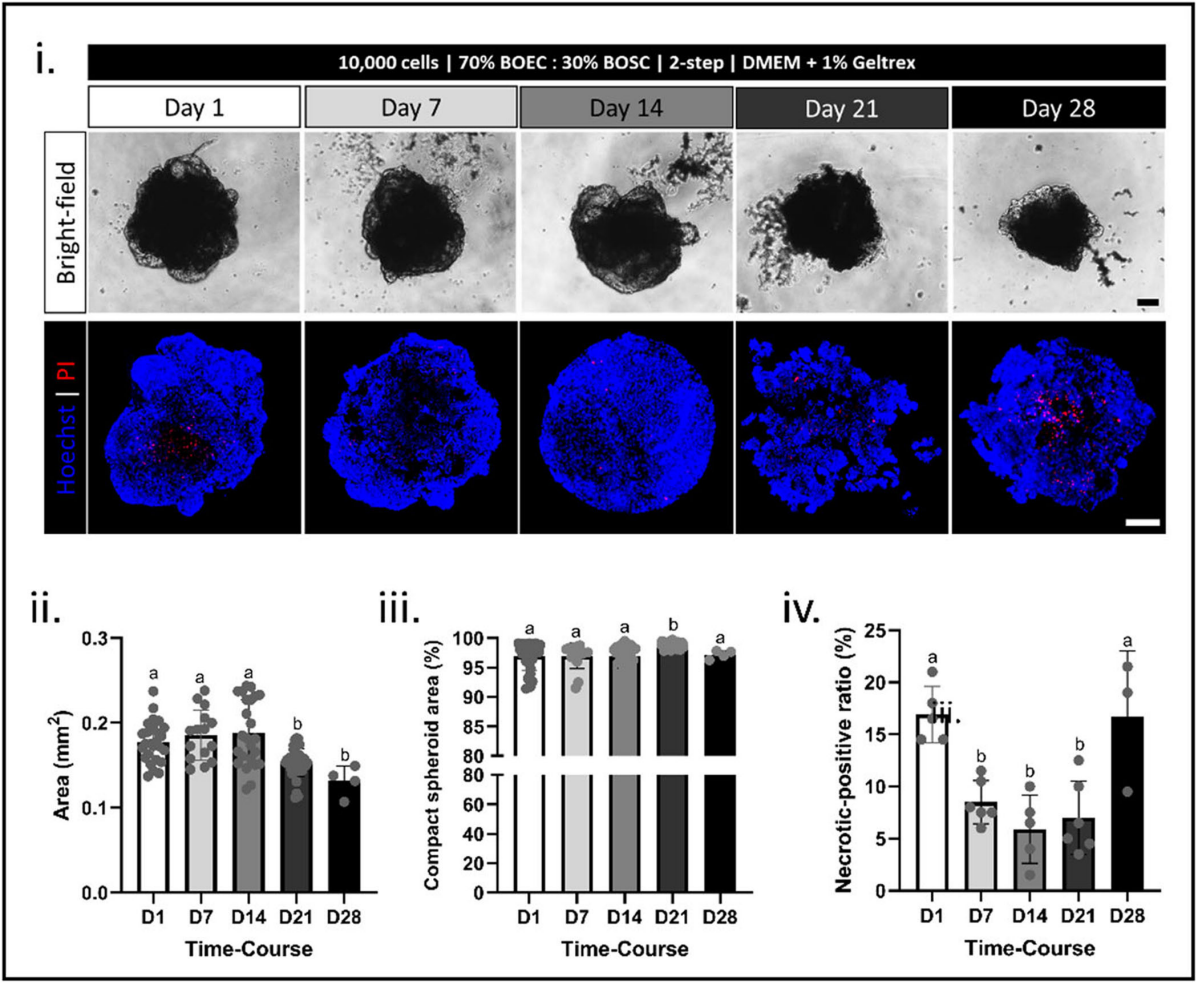
Long‐term spheroid viability evaluation: 10,000‐cell spheroids consisting of BOEC/BOSC‐mix (70/30%) were seeded as a two‐step protocol with culture media supplemented with 1% v/v Geltrex supplementation, cells were incubated in the supplemented media and on top of the Magnetic Bioprinting Plate for 72 h (considered day zero) and followed until Day 28 of culture. (i) representative images of spheroids (bright‐field and Hoechst + PI) on Days 1, 7, 14, 21, and 28 of culture, graphical results of (ii) spheroid area (mm^2^), (iii) spheroid compaction level (%), and (iv) necrotic‐positive cell ratio (%); different letters (a‐b) represent statistical differences (*p* < 0.05) among different culture days. All data are presented as mean ± standard deviation (SD) with single spheroid values represented in the graphs as a circle. All scale bars represent 100 μm. Abbreviations: BOEC, bovine oviduct epithelial cells; BOSC, bovine oviduct stromal cells; NS, NanoShuttle‐PL.

In Experiment V, the OMS's long‐term viability was evaluated until Day 28 of culture (Figure 4). Accessed on Days 1, 7, 14, 21, and 28, the OMS area was stable until Day 14 of culture, with a decrease in size on Days 21 and 28 (Figure 4‐i/ii). The compaction level was stable and always high (above 90%) throughout all 28 days of culture (Figure 4‐iii), supporting the spheroid structure stability, even when disturbed with a pipette. The necrotic‐positive cell ratio was higher on the first and last days of analysis (Day 1 and 28, respectively), but it remained low on Days 7, 14, and 21 (Figure 4‐i/iv). Similar to results observed in Experiment IV, this profile corroborates the theory that cells that did not survive the spheroid formation process (observed on Day 1) might be expelled from the OMS content between Day 1 and Day 7, remaining with a stable viability until Day 21. More investigation would be necessary to understand whether the increase in PI‐positive cell ratio observed on Day 28 is related to primary cell culture standard senescence or the involvement of other factors.

Even though other 3D culture systems support oviductal cell culture for longer periods (Chen et al. 2018; Kessler et al. 2015; Palma‐Vera et al. 2014), the OMS 21‐day culture is an outstanding achievement for a suspension culture system. Oviductal organoids (3D structures in which several organ‐specific cell types originated from a single stem cell) derived from human epithelial uterine tube cells have been cultured for at least 16 months (Kessler et al. 2015). When using BOEC, organoids were less efficient during culture, lasting 14 (Menjivar et al. 2023) or 21 days (Bourdon et al. 2021; Lawson et al. 2023). Still, successful long‐term BOEC culture has been achieved by using the insert culture system (a semi‐permeable membrane that supports mimicking the lumen and blood vessels conditions through, respectively, the apical and basal compartments, known as the air‐liquid interface (ALI)). BOEC were viable in the inserts for 10 days (Jordaens et al. 2015; Simintiras et al. 2016), three (Gualtieri et al. 2012, 2013), six (Palma‐Vera et al. 2014), or eight (Chen et al. 2017) weeks. Still, both these systems are classified as scaffold‐based 3D culture systems, which require physical support for cell self‐assembly, usually hydrogels for organoids (Kessler et al. 2015) and a porous membrane (polycarbonate or polyester) for inserts (Chen et al. 2013a, 2013b, 2017, 2018; Palma‐Vera et al. 2014). Adversely, the scaffold‐free systems (e.g., m3DB) challenge the long‐term culture. Explants, also known as worms, are everted vesicles (apical epithelial cell surfaces facing outward) of various sizes and shapes (100–1000 µm (Walter 1995)) formed by cell clumps within hours after starting culture, but viable for no more than 48 h (Gualtieri et al. 2013; Lefebvre et al. 1997; Rottmayer et al. 2006). When the suspension culture is extended (usually two more days), a cavity is formed in some of these oviductal explants, known as a vesicle‐like structure (Pranomphon et al. 2024; Xu et al. 1992). Although a ciliary movement is kept in these vesicle‐like structures for 10 days (Pranomphon et al. 2024; Xu et al. 1992), most structures progressively collapse throughout the culture, losing their cavity (up to 60% lost on Day 10 (Pranomphon et al. 2024)). The attachment/anchoring deprivation in scaffold‐free systems has been the main obstacle to long‐term culture (Meredith et al. 1993). On the other hand, the m3DB system, which supposedly stimulates ECM production within less than 30 min of culture (Tseng et al. 2013), supports cell‐matrix contact; this interaction suppresses cell death caused by the absence of this cell anchoring (Meredith et al. 1993). Thus, from Experiment V, we conclude that OMS is viable for 21 days in culture. This culture window period is highly relevant for studying the oviduct, especially for bovine species, since the estrous cycle is on average 21 days long.

### 2.6 Experiment VI: Evaluation of OMS Morphology and Cellular Organization

**Figure 5:**
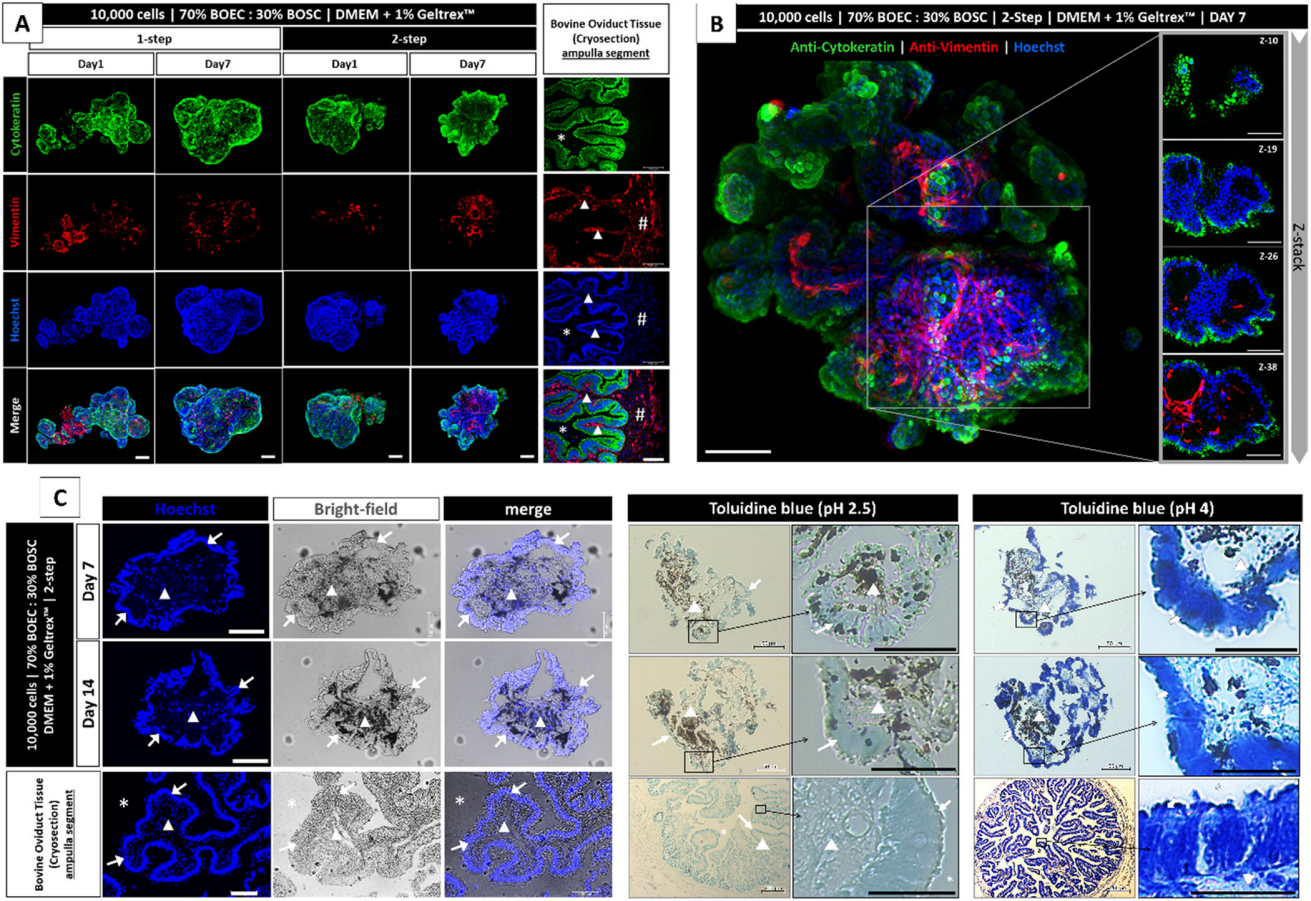
Characterization of the oviductal magnetic spheroid (OMS) structure organization. (A) Spheroids of 10,000‐cell size consisting of BOEC/BOSC‐mix (70/30%) were seeded as one‐ or two‐step protocols with culture media supplemented with 1% v/v Geltrex supplementation, cells were incubated in the supplemented media, and on top of the Magnetic Bioprinting Plate for 72 h, at this time the magnetic plate was removed and the culture media was changed (defined as day zero). Representative images of OMS at Day 1 and Day 7 from one‐ and two‐step protocols stained for anti‐cytokeratin (green, epithelial cell marker), anti‐vimentin (red, stromal cell marker), Hoechst 33342 (blue, DNA), and merged images (Maximum Intensity Z‐projection). The right panel represents the native oviductal tissue (cryosection of the ampulla segment) stained with the same markers. (B) Representative images from Supporting Information S6: Movie 1, Maximum Intensity Z‐projection (left panel) and selected z‐stacks (right panel) stained with anti‐cytokeratin, anti‐vimentin, and Hoechst, OMS at Day 7 from the two‐step protocol. (C) Spheroids of 10,000‐cell size consisting of BOEC/BOSC‐mix (70/30%) seeded as a two‐step protocol with culture media supplemented with 1% v/v Geltrex supplementation, cells were incubated in the supplemented media, and on top of the Magnetic Bioprinting Plate for 72 h, at this time the magnetic plate was removed, and the culture media was changed (defined as day zero). Representative images of paraffin sections of OMS at Day 7 (top panel) and Day 14 (middle panel), and a native oviductal tissue (bottom panel, ampulla segment). Samples were stained with Hoechst 33342 (DNA in blue, left panel), toluidine blue at pH 2.5 (middle panel), or toluidine blue at pH 4 (right panel). Scale bars are indicated in each picture; otherwise, white scale bars represent 100 μm, and black scale bars represent 20 μm. The symbols in the images indicate the oviductal lumen (*), stromal cell layer (arrowhead), epithelial cell layer (arrow), and muscular layer (#).

Concerning the OMS applicability as a 3D oviductal model, its similarity to the native tissue regarding morphology and cellular organization was assessed in Experiment VI. Using the one‐step (Figure S5‐iii) and two‐step (Figure S5‐iv) protocols, epithelial and stromal cell organization was analyzed by immunostaining (confocal). The one‐step protocol was included in this experiment to evaluate the influence of cell seeding order on the OMS's organization. As a result, on Day 1, BOEC and BOSC seemed randomly placed in a part of the OMS structure, as noticed by BOSC (red color) location (either in the one‐ or two‐step protocols, Figure 5A). On Day 7, the OMS organization was observed as a compartmentalized structure with the BOEC (cytokeratin‐positive) located on the OMS's periphery and the BOSC (vimentin‐positive) in the OMS's interior (Figure 5A,B, Supporting Information S6: Movie 1), no clear difference was observed when comparing one‐step versus two‐step (Figure 5A). In general, the native oviductal tissue has an epithelium organized as a pseudostratified columnar‐shaped cell. This epithelial cell layer is attached to the basement membrane, which separates the epithelium from the stroma layer. The latter consists of stromal cells, blood vessels, and other cell types organized in multilayers in the ECM (McDaniel et al. 1968), as observed in a native oviductal tissue transversal section (Figure 5A, right panel). The epithelial cell single‐layer facing the lumen (green color) is supported by the stroma cells (red color). Interestingly, we observed similar behavior in the OMS organization, as such the single BOEC layer facing the culture media in the OMS's periphery with the BOSC arranged in the OMS's interior (highlighted in the z‐stack images (Figure 5B) from the Supporting Information S6: Movie 1).

Each cell type organization also reflected its role in the tissue. As such, the epithelial cell layer might act as a selective barrier, made possible by an adjacent cell positioning, whereas the stroma layer plays a role in structuring and supporting other cells. As observed in a native oviductal tissue transversal section (Figure 5C, left‐bottom panel), the epithelial cell layer exhibits precisely aligned nuclei (arrows), and stromal cells are distributed in the submucosa layer (arrowhead). Compared to the native tissue, the OMS cultured for 7 or 14 days had a similar organization (Figure 5C, left‐top and left‐middle panels), an epithelial cell layer precisely aligned on the spheroid's outer layer (arrows), and dispersed stromal cells in the inner OMS's part (arrowheads). When submitted to toluidine blue staining, the OMS also presented a similar organization when compared to the native tissue (Figure 5C, middle and right panels). The blue toluidine at 2.5 pH represents only glycosaminoglycans. The cells showed a weak staining, both in the OMS and the native oviductal tissue (Figure 5C, middle panel). The same blue toluidine at 4 pH showed a more intensely stained, both in the OMS and the native oviductal tissue (Figure 5C, right panel), due to cytochemistry disclosure on DNA and RNA. No specific difference was observed regarding the spheroids' cytochemical analysis when comparing OMS from Days 7 and 14 of culture.

Interactions between epithelial and stromal cells are evident in the oviduct (Umezu and Tomooka 2004). Stromal cells are involved in the cell proliferation, cell fate, morphology, function, and hormonal response of epithelial cells (Brenner et al. 1990; Cunha et al. 1985; Haslam and Woodward 2003). Despite the evidence of stroma‐epithelial communication, the presence of stromal cells in the available 3D oviductal in vitro culture models has been limitedly explored. Reported by Simintiras and coworkers (Simintiras et al. 2016), the dual stroma‐epithelial culture was applied in the insert culture system, in which each cell type was placed on one side of the porous membrane, resulting in modulation by stromal cells in the formation of oviduct‐like fluid. Using the same 3D culture system, Chen and coworkers (Chen et al. 2013b) reported that the optimal polarized porcine oviduct epithelial cells are only achieved when cultured with fibroblast‐conditioned medium. Exploring a scaffold‐free system, Yamamoto and colleagues (Yamamoto et al. 2014) developed a spheroid of stromal cell aggregation. Although the stroma‐epithelial communication was absent, the spheroid model was more efficient in producing prostaglandin F2a compared to stromal cells in a monolayer system (Yamamoto et al. 2014). In our study, the OMS has the advantage of supporting the dual culture with direct contact of both cell types. Beyond the cell interaction, it was observed that the m3DB system encourages a refined cellular self‐organization in the OMS. The morphological similarities to the native oviductal tissue support the elaborated OMS organization, with emphasis on the cellular alignment, compartmentalization, and cytochemistry of glycosaminoglycans. The latter is highly relevant due to their involvement in providing structural and biochemical support to the cellular constituents of biological tissues (Mattson et al. 2019).

Hence, we conclude from Experiment VI that BOEC and BOSC are capable of self‐organizing in the OMS by Day 7 of culture. Such organization reflects in a high similarity between the OMS and the native oviductal tissue architecture, highlighted by: (i) compartmentalization of epithelial and stromal cells in the OMS, (ii) epithelial cells organized as a single layer, (iii) apical side of epithelial cells in contact with the environment (culture media) like the oviductal epithelium is in contact with the lumen, and (iv) the stromal cells providing the support for the epithelial cells.

### 2.7 Experiment VII: Evaluation of Cell Ciliation and Secretory Marker in the OMS

**Figure 6:**
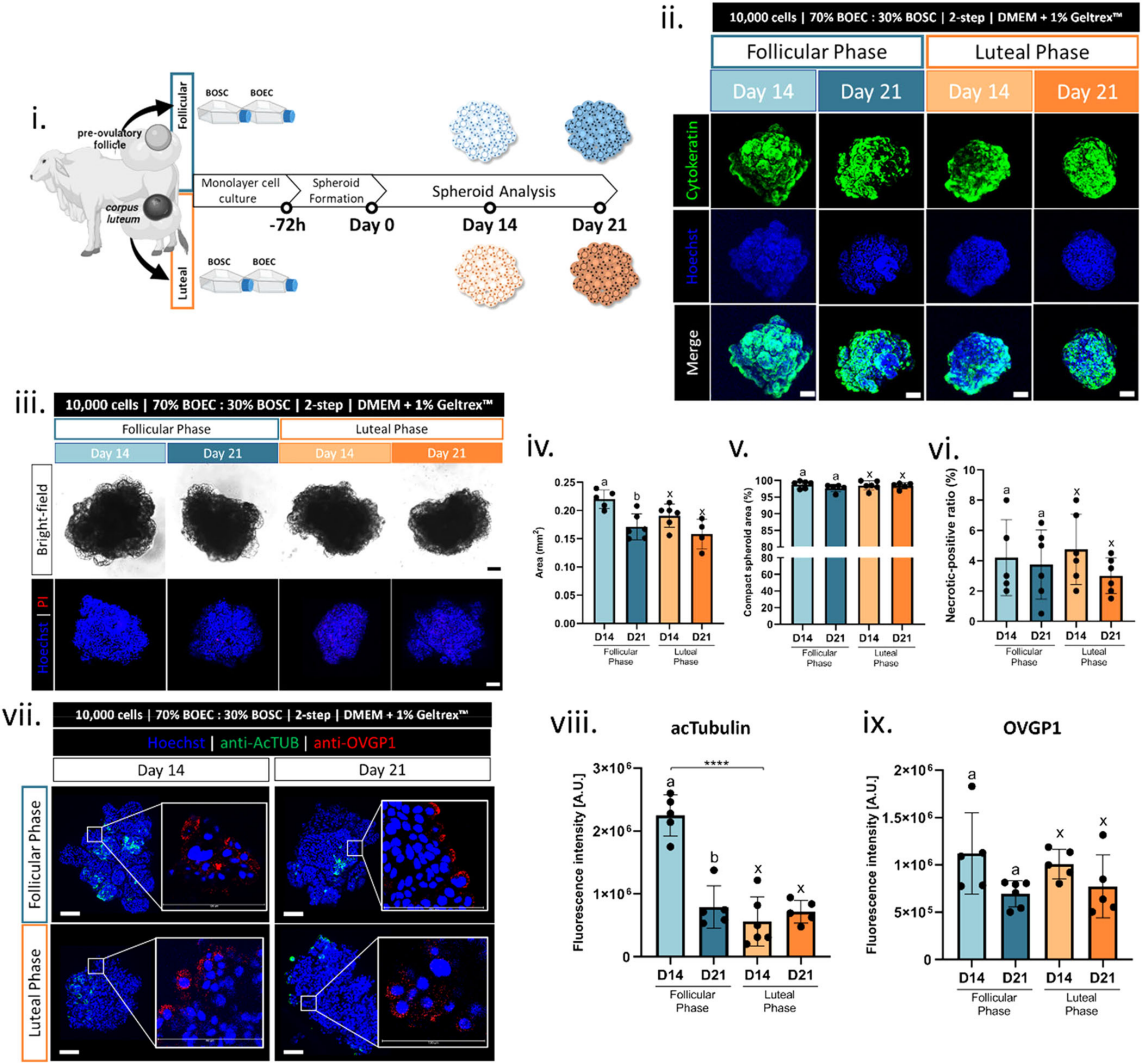
Characterization of cell ciliation and secretion markers in OMS formed by cells harvested at different estrous cycle phases. OMS formed by BOEC and BOSC harvested from oviductal tissues at follicular or luteal phases of the estrous cycle and cultured for 14 or 21 days (i) representation of the experimental design of OMS formation and culture, (ii) representative images of OMS stained with anti‐cytokeratin (green, epithelial cell marker) and Hoechst 33342 (blue, DNA) and merged images, (iii) representative images of OMS in bright‐field and co‐stained with Hoechst 33342 (nuclei) and Propidium Iodate (PI, necrotic‐positive cell marker), graphical results of (iv) spheroids area (square millimeter, mm^2^), (v) spheroid compaction level (percentage, %), and (vi) necrotic‐positive cell ratio (%), (vii) representative images of OMS stained with anti‐acetylated alpha‐tubulin (acTUB, green, cilia marker), anti‐oviduct‐specific glycoprotein 1 (OVGP1, red, secretion marker), and Hoechst 33342 (blue, DNA) and merged images, low and high magnifications (inserts) and graphical results of relative fluorescent intensity of (viii) acTUB and (ix) OVGP1 presented as arbitrary unit (A.U.) of the specific pixel quantification per spheroid area; different letters represent statistical differences (*p* < 0.05) among different days of culture in OMS from follicular (a‐b) and luteal (x‐y) phases and **** represents statistical difference (*p* < 0.0001) when comparing follicular versus luteal phases. All data are presented as mean ± standard deviation (SD) with single spheroid values represented in the graphs as a circle. Scale bars represent 100 μm (white bars) or 50 μm (gray bars). Abbreviations: BOEC, bovine oviduct epithelial cells; BOSC, bovine oviduct stromal cells.

Beyond the morphological organization, the native oviduct epithelial cells are highly polarized and composed of ciliated and non‐ciliated cells (Yániz et al. 2000). Yet, the maintenance of such cellular features is a challenge for the in vitro systems. Known as cell dedifferentiation, once removed from the in vivo conditions, the oviductal epithelial cells progressively lose morphology features, like the columnar shape, cell polarity, and cell ciliation/secretory granules (Chen et al. 2013a; Danesh Mesgaran et al. 2016; Rottmayer et al. 2006; Schmaltz‐Panneau et al. 2014). In some 3D culture systems, such as the worms and vesicle‐like structures, this cell dedifferentiation process is delayed by maintaining the morphological features for up to a few days (2–10 days) (Mahé et al. 2023; Pranomphon et al. 2024; Xu et al. 1992). Distinctively, other systems are known to stimulate cellular redifferentiation after the dedifferentiation process from the monolayer cell culture. It usually occurs by transferring the dedifferentiated cells to a 3D culture system that can re‐establish morphological and functional cellular features, such as organoids (Chen et al. 2017; Kessler et al. 2015; Lawson et al. 2023; Palma‐Vera et al. 2014) and inserts‐ALI (Chen et al. 2013a, 2013b, 2017, 2018; Ferraz et al. 2018; Palma‐Vera et al. 2014). In our study, after the monolayer cell culture, the OMS consisting of BOEC/BOSC‐mix presented a cellular morphological organization with patterns in vivo‐like (Experiment VI). Hence, in Experiment VII, we evaluated if this structural organization would support cell redifferentiation, including cell ciliation and secretion markers. The acetylated alpha‐tubulin (acTUB) was selected to identify the ciliation, while the expression of oviduct‐specific glycoprotein 1 (OVGP1) was selected as the marker for cell secretion. Due to the influence of the estrous cycle on cell ciliation and secretion in native tissues (Barton et al. 2020; Binelli et al. 2018; McDaniel et al. 1968), the OMS was formed with cells from two estrous cycle phases (follicular and mid‐luteal phases) and cultured for 14 or 21 days (Figure 6‐i).

As a result, the OMS morphology organization was similar among the groups: a single epithelial cell layer on the OMS periphery (either on Day 14 or 21, Figure 6‐ii). The spheroid's area had no effect from the cell source (follicular vs. luteal), but OMS from Day 21 was smaller than from Day 14 of the follicular phase (Figure 6‐iii/iv). The OMS stability (compact level, Figure 6‐iii/v) and viability (necrotic‐positive cell ratio, Figure 6‐iii/vi) showed no effect of cell source (follicular vs. luteal) or culture day (14 vs. 21). Regarding the cell differentiation markers, the acTUB‐positive structures (cell ciliation) were located in the outer region of the spheroids (Figure 6‐vii), indicating that the spheroids conserve their apical‐basal polarity with the apical cell facing outwards (culture media). Corroborating with that, the OVGP1‐positive structures (secretion marker) were mainly on the spheroid surface (Figure 6‐vii), as the secretion granules are most frequent in the apical parts of the cells in the native tissue (Bjorkman and Fredricsson 1961). However, the relative OMS area covered by acTUB‐positive regions was limited in all the experimental groups, without elongated cilia, indicating an absence of mature secondary cilia in the OMS (Figure 6‐vii). Moreover, when the quantitative analysis was performed, the relative levels of acTUB were higher on Day 14 than Day 21 in OMS from the follicular phase and higher in OMS from the follicular than luteal phase on Day 14 (Figure 6‐viii). The low cell ciliation with a decrease when further in culture (D14 > D21, Figure 6‐viii) goes in the opposite direction when compared to the ALI system, which demonstrated that the cellular markers related to redifferentiation increase longer in culture (Palma‐Vera et al. 2014; Zhu et al. 2020). On the contrary, the relative levels of OVGP1 were similar among the groups (Figure 6‐ix). The OVGP1 expression in the OMS is a remarkable achievement since it is a well‐known example of the loss marker, which is promptly downregulated under in vitro conditions and cannot be triggered by ovarian steroids anymore (Briton‐Jones et al. 2002; Danesh Mesgaran et al. 2016; Schoen et al. 2008). Therefore, from Experiment VIII, we conclude that OMS supports cellular redifferentiation, including cell ciliation (although partially limited) and OVGP1 expression. Moreover, the estrous cycle phase when BOEC and BOSC were collected had a slight effect on the outcome parameters.

### 2.8 Experiment VIII: Evaluation of OMS Responsiveness for Ovarian Steroid Hormone

**Figure 7:**
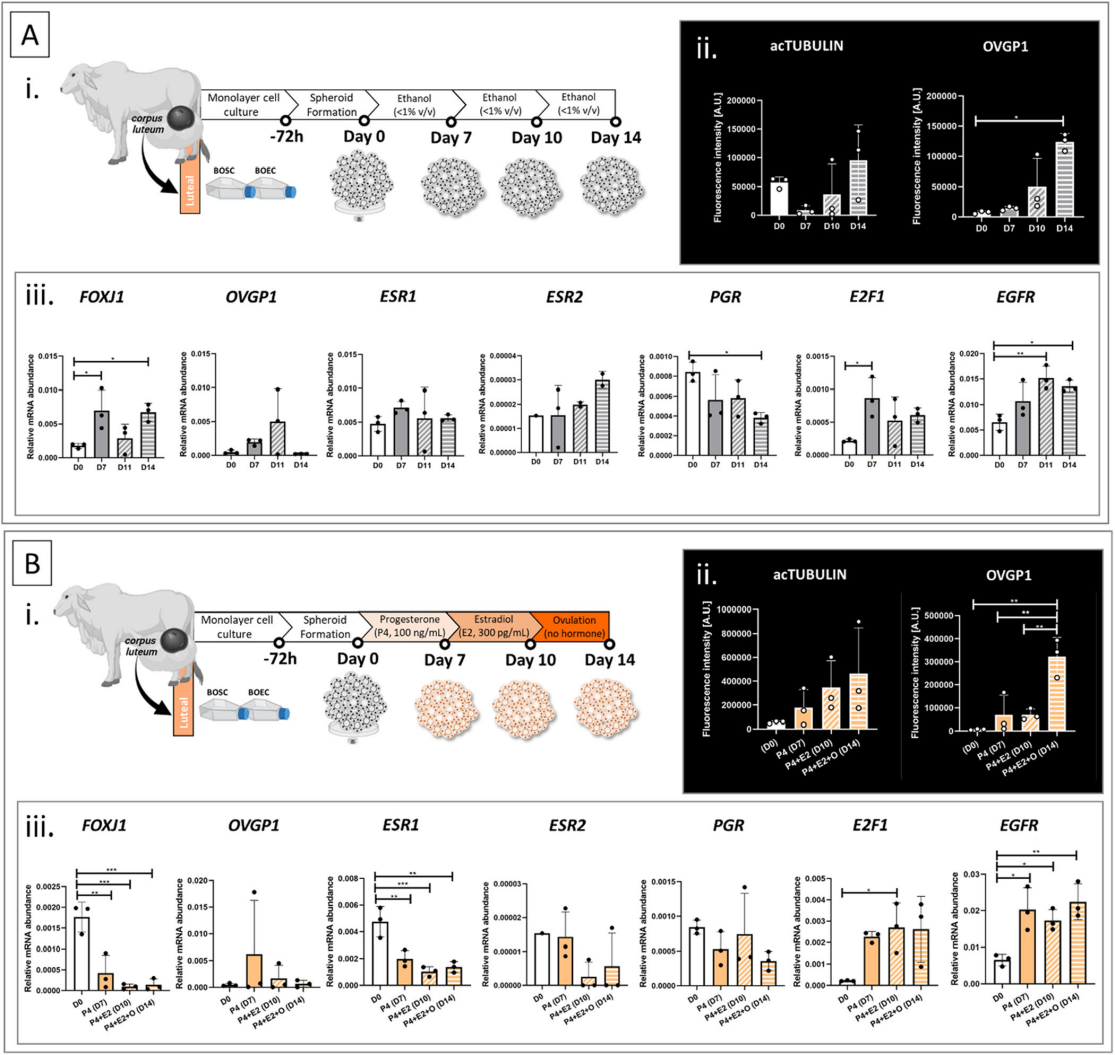
Hormone responsiveness of OMS. 10,000‐cell spheroids of BOEC/BOSC‐mix (70%/30%) were formed using the two‐step protocol. OMS was formed with cells harvested during the luteal phase of the estrous cycle and evaluated during different days of culture or submitted to hormonal treatment. (A) OMS from different culture days, not treated, and (B) OMS submitted to hormone treatment: progesterone (P4, 100 ng/mL) for seven days to mimic a luteal phase, followed by estradiol (E2, 300 pg/mL) for three days to mimic a follicular phase, followed by four days with no hormone in the media, simulating the post‐ovulatory period. (i) Schematic representation of the experimental designs; (ii) AcTUB and OVGP1 protein quantification (fluorescence intensity per area, presented as an arbitrary unit, A.U.); * and ** represent statistical difference (*p* < 0.05 and < 0.01, respectively). (iii) Relative mRNA abundance of the genes forkhead box protein J1 (*FOXJ1*, ciliation marker), oviduct‐specific glycoprotein 1 (*OVGP1*, secretion marker), estrogen receptor 1 and 2 (*ESR1* and *ESR2*, respectively), progesterone receptor (*PGR*), E2F Transcription Factor 1 (*E2F1*), and Epidermal Growth Factor Receptor (*EGFR*), presented as an arbitrary unit (A.U.); *, **, and *** represent statistical difference (*p* < 0.05, < 0.01, and < 0.001, respectively). All data are presented as mean ± standard error (SE) with single values represented by the black circles.

As mentioned before, the oviductal cell ciliation and secretion activity are controlled by the ovarian steroid hormone throughout the estrous cycle phases (Barton et al. 2020; Binelli et al. 2018; McDaniel et al. 1968). Hence, we decided to put the OMS under a hormonal treatment mimicking the estrous cycle phases as a proof of concept. Because in Experiment VII the Day 14 OMSs showed a bigger area than Day 21 (Figure 6‐iv), the hormone treatment was performed for 14 days in total. Because the cell ciliation (acTUB positive structure) decreased throughout time in spheroids produced with cells from the follicular phase, in the next experiment, only cells harvested at the luteal phase were used. In Experiment VIII, the OMSs (10,000‐cell spheroid, 70%BOEC/30%BOSC, 1% v/v Geltrex supplementation, two‐step protocol, cells harvested at luteal phase) were hormone‐treated for 14 days in total. First, OMSs were submitted to 14 days with dilutor ( < 1% v/v absolute ethanol), as a control condition (Figure 7A). To mimic the estrous cycle stages, OMSs were submitted to 7 days of luteal phase simulation (100 ng/mL P4), followed by 3 days of follicular phase simulation (300 pg/mL E2), and finally, 4 days of post‐ovulatory simulation (absence of hormone treatment), totaling 14 days of culture (Figure 7B). The hormone concentrations were designed based on the local bovine oviductal levels, which can be up to 50 times higher than the circulatory blood levels (Lamy et al. 2016). OMSs were analyzed after luteal phase simulation (Day 7), after follicular phase simulation (Day 10), and after post‐ovulatory phase simulation (Day 14) in the hormone‐treated groups (Figure 7B). The control group (without hormone treatment) was included to evaluate if the effect on the outcomes was due to the OMS hormone responsiveness (Figure 7B) or due to the days of culture (Figure 7A).

As a result, the acTUB protein levels were unaffected by the culture days (Figure 7A‐ii) or hormone treatment (Figure 7B‐ii). Distinctively, the OVGP1 protein levels were significantly higher in untreated OMS on day 14 when compared to Day 0 (Figure 7A‐ii). In the treated OMS, the OVGP1 protein levels were the highest at post‐ovulatory simulation when compared to the time zero (D0), luteal phase simulation, and follicular phase simulation (Figure 7B‐ii). Interesting, the OVGP1 expression has been described of being estrous cycle‐dependent (Buhi 2002). The inhibition of OVGP1 expression during the luteal phase simulation (Day 7) is corroborated by previous studies, which show that progesterone antagonized the effect of estradiol, suppressing OVGP1 expression (Buhi 2002; Ito et al. 2016; Kavanaugh et al. 1992; Killian et al. 1989).

To gain insight into the OMS hormone responsiveness, the OMSs were submitted to mRNA relative abundance evaluation. Five genes related to the oviductal function were analyzed: Forkhead box protein J1 (*FOXJ1*, ciliation marker), *OVGP1*, estrogen receptor 1 and 2 (*ESR1* and *ESR2*, respectively), and progesterone receptor (*PGR)* (Bauersachs et al. 2004; Binelli et al. 2018; Cerny et al. 2015; Lamy et al. 2016). In addition, two genes related to cell division were quantified as indicative of cell proliferation (E2F Transcription Factor 1 (*E2F1*) and Epidermal Growth Factor Receptor (*EGFR*)) (Ertosun et al. 2016; Nikpey et al. 2018). As a result, three genes presented a distinct pattern in the nontreated groups (different culture days, Figure 7A‐ii) compared to hormone‐treated groups (Figure 7B‐iii). Specifically, the *FOXJ1* levels increased during culture time (Figure 7A‐iii), while they were decreased by hormone treatment (Figure 7B‐iii). The *ESR1* levels were unaffected by the culture time (Figure 7A‐iii), while they decreased by hormone treatment (Figure 7B‐iii). Last, the *PGR* levels were downregulated after culture time (Figure 7A‐iii), while they were unaffected by hormone treatment (Figure 7B‐iii). In other words, the abundance of these genes has been differently modulated through culture time, if the OMSs were hormone‐treated during culture. Indeed, it has been reported that the abundance of these genes is estrous cycle‐dependent (Binelli et al. 2018). As previously reported, both cell ciliation and secretion activity are known to decrease in the native oviduct during the P4‐dominant phase (the luteal phase), while the proportion of ciliated cells and the oviductal fluid secretion reach the highest levels during the peri‐ovulatory period (E2‐dominant period) (Binelli et al. 2018; Ferraz et al. 2018; Ito et al. 2016; Kavanaugh et al. 1992). Moreover, P4 treatment has been demonstrated to downregulate the expression of its receptor (PGR), as well as E2 receptor (*ESR1*); while the E2 treatment has the opposite effect, increasing the *PGR* and *ESR1* levels (Chen et al. 2013a, 2018). Based on the results of Experiment VIII, we suggest that the OMSs have been modulated by the P4 treatment (Day 7), which caused the downregulation of *FOXJ1* and *ESR1* levels compared to Day 0 (Figure 7B‐iii). However, it seems that E2 treatment (Day 10‐14) has not been sufficient to increase the abundance of such genes. Such limitation on the effect of E2 treatment can be related to the E2 concentrations explored in our study, since E2 and P4 effects work in a dose‐dependent manner (Chen et al. 2018). Another hypothesis is that the peri‐ovulatory milieu consists not only of E2 and P4; for instance, the preovulatory luteinizing hormone surge, together with increasing E2 secretion from the preovulatory follicle, and basal P4 levels from the regressing corpus luteum is necessary to induce modulation in the oviductal cells (Wijayagunawardane et al. 2005). Last, but not least, the abundance of *E2F1* and *EGFR* increased throughout time culture (Figure 7A‐ii) and hormone‐treated OMS (Figure 7B‐iii) compared to Day 0. Indicative of cell proliferation (Ertosun et al. 2016; Nikpey et al. 2018), the higher abundance of these genes after spheroid formation corroborates the OMS long‐term survival in our study.

## 3 Conclusion

In this study, we have described a 3D scaffold‐free culture system that supports cellular self‐organization, composed of both oviductal epithelial and stromal cells, and capable of re‐establishing some cell redifferentiation markers. The OMS recapitulated many of the oviductal tissue structural features of a simple epithelial cell layer, arranged as adjacent cells, and positioned above the stromal cell arrangement. Such spheroid architecture supported the coordination of apical‐basolateral polarity of epithelial cells, which resulted in cell redifferentiation by expressing cilia and secretion markers. Moreover, because it is a scaffold‐free 3D culture system, the OMS can be easily handled using a pipette tip. Last, the epithelial cells are located in the spheroid's periphery with the apical part facing outwards, therefore, they are directly accessed. These remarkable achievements open many possibilities for the applicability of the OMS as a model to study oviductal physiology and its roles in the reproductive field.

As expected from an in vitro culture model, the OMS has its advantages and limitations. The OMS successfully supported the coculture of different tissue cell types (epithelial and stromal), which represents a model more similar to the complex oviductal tissue architecture, as well as allows the cell‐cell structural and functional interactions. Moreover, the different cell types can be arranged in different proportions during OMS formation along with differential spheroid cell number sizes, which are assured by the one‐to‐one system: one well, one spheroid formation. The one‐to‐one system also secures a uniform spheroid size and shape, and high spheroid formation repeatability. Last, but not least, the relative 21‐day long‐term culture, associated with a scaffold‐free system and the apical epithelial cell polarity facing outwards, facilitates the OMS handling, experimental treatments, and future coculture with embryos and/or gametes. The latter advantage is also related to a limitation since the same culture media would be applied for the OMS and embryos and/or gametes, unable to mimic the compartmentalized organization of the native tissue. Another disadvantage of this system is the need to submit the cells for a monolayer culture before spheroid formation, which leads to cell dedifferentiation, a longer experimental protocol (time‐consuming), and the need for enzymatic treatment for cell detachment from the culture flask. Eventually, some limitations were also introduced specifically in this study, such as the use of cells collected from animals from a slaughterhouse, which adds an extra bias due to the scarce information regards the animals, as well as the estrous cycle phase identification by ovarian morphology, which is not fully precise. Not to mention, the BOEC‐only spheroid could not be standardized in this study. Regarding the hormone treatment, OMS seemed to well respond to P4 treatment, however, E2 treatment was insufficient to arouse hormone responsiveness in the OMS, which could also be related to the low number of spheroids in some analyses.

Although it might be relevant only for a specific researcher's group, it is worth mentioning that this study was performed entirely at a Brazilian Federal University, where the acquisition of specific equipment is highly costly (due to the currency devaluation compared to the Dollar and Euro) and very time‐consuming (due to the bureaucracy and delivery time to import equipment). Thus, the standardization of the m3DB system as a model to culture oviductal cells is exceptionally relevant, since it requires the m3DB kit (low attachment culture plate, Nanoshuttle‐PL, and magnetic plate), a centrifuge, and a CO2 cell incubator. In other words, our study contributes to turning the 3D cell culture research field into more easy/open access and possible to replicate, including third‐world country conditions, which we consider to be very relevant for truly global research and equitable knowledge circulation.

All things considered, the development of the OMS as a model for studying the oviduct tissue possesses the potential to enhance the understanding of oviductal and reproductive physiology not only in bovine species but also in other species, such as humans. In vitro models, like the OMS, offer a practical alternative to bridge the gap between ethical issues and experimentation in animals. This groundbreaking oviductal model might serve as a platform to study oviductal function, maternal‐embryo communication, and gamete capacitation. Escalating the complexity of this model, future studies might incorporate more cell types other than epithelial and stromal cells, genetically modified/edited cells, and pathological compounds, aiming to apply the OMS as an approach to test therapies and technologies to improve assisted reproduction success.

## 4 Materials and Methods

All the reagents were from Sigma‐Aldrich/Merck otherwise described. All the cell and spheroid cultures were performed in a 5% CO_2_ incubator at 38.5°C and high humidity in an atmosphere. The Ethics Committee approved all experimental procedures involving animals on the Use of Animals of the Federal University of ABC (UFABC).

### 4.1 Experimental Design

This study was carried out in eight experiments using bovine oviductal epithelial cells (BOEC) and stromal cells (BOSC):

Experiment I: Evaluation of cell number size per spheroid. Spheroids consisting of BOEC‐only (100%BOEC) or BOEC/BOSC‐mix (70%/30%) were cultured for 48 h in the magnetic bioprinting plate as 50,000, 25,000, 10,000, and 5000 cells per spheroid.

Experiment II: Evaluation of cellular magnetization methods. BOEC and BOSC were magnetized by overnight incubation or centrifugation. Spheroids consisting of BOEC‐only (100%BOEC) or BOEC/BOSC‐mix (70%/30%) were cultured for 48 h in the magnetic bioprinting plate as 10,000‐cell per spheroid.

Experiment III: Evaluation of cell seeding protocols and cell proportion (BOEC: BOSC). For spheroid formation, BOEC and BOSC were seeded in the well at the same time (one‐step protocol) or the BOEC was seeded 24 h after BOSC being seeded (two‐step protocol). Spheroids consisting of BOEC‐only (100%BOEC) or BOEC/BOSC‐mix (70%/30% or 50%/50%) were cultured as 10,000‐cell for 48 h in the magnetic bioprinting plate, followed by culture without the magnetic bioprinting plate until 144 h of culture.

Experiment IV: Evaluation of culture media supplementation with ECM. For spheroid formation, BOEC and BOSC were seeded using the one‐ or two‐step protocol, in culture media supplemented with Geltrex (0, 0.5, 1, and 2% v/v). 10,000‐cell spheroids consisting of BOEC/BOSC‐mix (70%/30% or 50%/50%) were cultured for 48 h in the magnetic bioprinting plate, following in culture without it and in the absence of Geltrex (until 216 h).

Experiment V: Evaluation of long‐term culture of Oviductal Magnetic Spheroids (OMS). For spheroid formation, BOEC and BOSC were seeded using the two‐step protocol. 10,000‐cell spheroids consisting of BOEC/BOSC‐mix (70%/30%) were cultured in media supplemented with 1% v/v Geltrex for 72 h on top of the magnetic bioprinting plate. Next, the magnetic bioprinting plate was removed, and Geltrex‐free media was placed (defined as Day Zero). Thereafter, spheroids were cultured for 1, 7, 14, 21, and 28 days.

Experiment VI: Evaluation of OMS morphology and cellular organization. BOEC and BOSC were seeded as 10,000‐cell spheroids consisting of BOEC/BOSC‐mix (70%/30%) using the one‐ and two‐step protocol, in culture media supplemented with 1% v/v Geltrex. After 72 h, the magnetic bioprinting plate was removed, and Geltrex‐free media was placed, defined as Day Zero. Spheroids were stained for epithelial (anti‐cytokeratin [CYT]), stromal (anti‐vimentin [VIM]), and nuclei (Hoechst 33342) markers on Days 1 and 7.

Experiment VII: Evaluation of cell ciliation and secretory marker in the OMS. BOEC and BOSC harvested from oviductal tissues of cows at follicular or luteal phases of the estrous cycle were used for OMS formation. OMSs (10,000‐cell, BOEC/BOSC‐mix (70%/30%), two‐step protocol with 1% v/v Geltrex media supplementation) were stained for cilia marker (anti‐acetylated alpha‐tubulin [acTUB]), secretion (anti‐oviduct‐specific glycoprotein 1 [OVGP1]), and nuclei (Hoechst 33342) markers on Day 14 or 21 (Day 0 was defined as the day of magnetic bioprinting plate was removed and Geltrex‐free media was placed).

Experiment VIII: Evaluation of OMS responsiveness for ovarian steroid hormone. BOEC and BOSC harvested from oviductal tissues of cows at luteal phase of the estrous cycle were used for OMS formation. OMSs (10,000‐cell, BOEC/BOSC‐mix (70%/30%), two‐step protocol with 1% v/v Geltrex media supplementation) were submitted to hormonal treatment mimicking the estrous cycle phases, or not (control). Seven days of luteal phase simulation (100 ng/mL progesterone [P4]), followed by 3 days of follicular phase simulation (300 pg/mL estradiol [E2]), and finally, 4 days of post‐ovulatory simulation (absence of hormone treatment), totaling 14 days of culture. OMSs on days 0, 7, 10, and 14 were analyzed for protein expression (stained for cilia marker (anti‐acetylated alpha‐tubulin), secretion (anti‐oviduct‐specific glycoprotein 1), and nuclei (Hoechst 33342)) and mRNA abundance. Day 0 was defined as the day of magnetic bioprinting plate was removed and Geltrex‐free media was placed.

In all experiments, the analyses were performed in at least three spheroids in each experimental group.

### 4.2 Oviductal Cells Collection and Isolation

Reproductive tracts from cows were collected at a local slaughterhouse. The tissues were collected immediately after slaughter ( ~ 15 min) and were transported to the laboratory on ice within 2 h. Based on ovarian morphology (Ireland et al. 1980), the estrous cycle phases were classified post‐mortem in:

A. Follicular (follicular phase: estimated to be between 18 and 20 days of the estrous cycle), when a Corpus Luteum with less than 1 cm in diameter, light yellow/white external colour, and the yellow internal colour was present, in addition to the presence of a preovulatory follicle more than 10 mm in diameter;
B. Luteal (mid‐luteal phase: estimated to be between 5 and 10 days of the estrous cycle), when a Corpus Luteum with more than 1.6 cm in diameter, red/brown color in the apex only and orange colour in the remaining portion was present, in addition to the presence of a dominant follicle more than 10 mm in diameter.

Twelve animals were used in this study (six of each estrous cycle stage). The ipsilateral oviducts of each animal (ipsilateral to the pre‐ovulatory follicle for the follicular phase and ipsilateral to the Corpus Luteum for the luteal phase) were dissected free of surrounding tissue. The tubular segment of oviducts (from isthmus until ampulla) was quickly washed in 70% ethanol, followed by three washes in phosphate‐buffered saline (PBS) supplemented with 100 U/mL penicillin, 100 µg/mL streptomycin, 100 µg/mL gentamycin, and 2.5 µg/mL amphotericin B (room temperature). The length of the total tubular segment of the oviducts was measured; only oviducts within a minimal 20 cm were used in this study. No difference was observed in the oviduct length when comparing the estrous cycle phases (Follicular: 21 ± 1.8 cm; Luteal: 21.5 ± 1.9 cm, *p* > 0.05). Next, the BOEC and BOSC isolations were processed, as described below.

#### 4.2.1 BOEC Collection and Isolation

Only BOECs from the ampulla segment were collected. For that, the ampulla segment was mechanically squeezed with tweezers, cells were pooled in warm (37°C) HEPES buffered Medium 199 supplemented with 100 U/mL penicillin, 100 µg/mL streptomycin, 100 µg/mL gentamycin, and 2.5 µg/mL amphotericin B (Base Medium—BM), and centrifuged at 200*g* for 5 min. The supernatant medium was discarded, and the cells were resuspended in 2 mL of warm (37°C) 1X Trypsin diluted in PBS and incubated for 3 min at 38.5°C. Next, the enzyme activity was inactivated by adding 4 mL of BM supplemented with 20% v/v fetal bovine serum (FBS). Cells were mixed using a 21 G needle to break the cellular clumps, followed by two rounds of centrifugation at 200*g* for 5 min with 3 mL BM supplemented with 10% v/v FBS. Cells were placed in a 25 cm^2^ flask (T25) and incubated for 2 h in BM supplemented with 10% v/v FBS for fibroblast exclusion by time adhesion (Davidoff et al. 2015) to the plate (defined as pre‐culture). After incubation, the nonattached cells were recovered, centrifuged (200*g* for 5 min), counted, and frozen as 10^6 ^cell/mL solution (1 mL/tube) using the Mrs. Frosty system (Thermo Scientific) in HEPES buffered Medium 199 supplemented with 20% v/v FBS and 10% v/v dimethyl sulfoxide (DMSO). After 4 h at −80°C, the cryotubes were removed from Mrs. Frosty and kept at −80°C until use. The BOEC were processed and stored individually per animal. The epithelial cell population purity was confirmed using an antibody anti‐cytokeratin (a specific epithelial cell marker), performed as described below and presented in the results session.

#### 4.2.2 BOSC Collection and Isolation

After mechanically squeezing, the remaining oviductal segment was longitudinally opened, and pieces of 0.25 cm^2^ of tissue were cut for BOSC isolation. The tissue fragments were placed in a 6‐well plate with the luminal part upwards and covered with a glass slide. After 2 min of rest for the tissue attachment, 3 mL BM supplemented with 10% v/v FBS was added per well. Media was replaced every other day for 10–12 days when a significant number of stromal cells were visible growing around the tissue fragment. At this point, the stromal cell identification was performed by morphological parameters. On days 10–12 of culture, the tissue fragment was discarded, and cells were detached using trypsin (1X) for 2 min at 37°C (a short time was essential to avoid BOEC as much as possible). After centrifugation (200*g* for 5 min), the cell pellet was resuspended, seeded in a new T25 plate, and incubated for 10 min. Next, the nonattached cells were removed to eliminate as many BOEC as possible by time adhesion (Davidoff et al. 2015). The attached cells were cultured until reaching 80% confluence, with BM supplemented with 10% v/v FBS changed every other day. In case of BOEC contamination, the flask was resubmitted to the trypsin procedure (2 min trypsin incubation and 10 min cell attachment). Most of the BOSC population was purified within two passages, with some samples requiring three passages. Once purified, the BOSC were pooled per estrous cycle phase (follicular or luteal phase) by mixing cells of six animals and frozen using the Mrs. Frosty system (Thermo Scientific) as 10^4^ cell/mL solution (1 mL/tube) in HEPES buffered Medium 199 supplemented with 20% v/v FBS and 10% v/v DMSO. After 4 h at −80°C, the cryotubes were removed from Mrs. Frosty and kept at −80°C until use. The stromal cell population purity was confirmed using an antibody anti‐vimentin (a specific stromal cell marker), performed as described below and presented in the results session.

### 4.3 Oviductal Cells Thawing and Culturing

Before starting 3D spheroid cultures, cells (BOEC and BOSC) were cultured as monolayers (2D). All the 2D cultures of BOEC and BOSC were done in separate flasks. Cells were thawed in a water bath (37°C for 1–2 min). The BOEC solution (10^6^ cells/mL) was transferred to a 15 mL tube, diluted in 5 mL BM supplemented with 10% v/v FBS, and centrifuged (200*g* for 5 min). The cell pellet was resuspended in 7 mL BM supplemented with 10% v/v FBS and transferred to a T25. The BOSC solution (10^4^ cells/mL) was transferred to a T25 with 5 mL BM supplemented with 10% v/v FBS. After 30 min, the media was replaced with fresh 7 mL BM supplemented with 10% v/v FBS. Culture media was replaced every other day until the cells reached 80% confluence. BOEC was cultured as a pool of at least four animals (two follicular and two luteal phases) for experiments I–VI. For experiment VII, the BOEC was cultured separately by the animal (six animals from each estrous cycle phase, follicular and luteal). For experiment VIII, only cells from the luteal phase were used. BOSC from different animals were pooled before freezing, separated by the estrous cycle phase (follicular or luteal). For experiments I‐VI, the BOSCs from both follicular and luteal phases were mixed. For experiments VII and VIII, the BOSC from each estrous cycle phase was associated with the BOEC of the same phase.

### 4.4 Oviductal Magnetic Spheroid Culture

Oviductal spheroids were initially cultured following the manufacturer's instructions, with modifications explored throughout this study. The initial protocol consisted of rinsing the BOEC and BOSC growing as a monolayer (Section 4.3) with warm PBS (three times). Subsequently, cells were detached from the culture flask using trypsin (1X), centrifuged (200*g* for 5 min), and the cell pellet was resuspended in BM supplemented with 10% v/v FBS. After counting each cell type, the specific number of cells required for each experiment was placed in a 1.5 mL centrifuge tube. The media volume was adjusted to 1 mL, and NanoShuttle (NS) was added (1 μL per 10.000 BOEC and 1 μL per 10.000 BOSC—Experiments I–III—or 0.5 μl per 10.000 BOSC – Experiment IV‐VIII). This solution was submitted to three rounds of centrifugation (500*g* for 5 min) with homogenization by pipetting up and down between cycles. After the third cycle, the media was replaced by Spheroid Culture Media (SCM, consisting of DMEM (Gibco BRL, Paisley, UK, Cat. A1443001), no phenol red due to estrogenic activity (28), supplemented with 5% FBS, 0.18 mM glutamine, 2.74 mM glucose, 0.12 mM pyruvate, 5 µg/mL insulin, 5 µg/mL transferrin, 25 ng/mL epidermal growth factor, 50 µg/mL gentamycin, 0.125 µg/mL amphotericin B, 100 U/mL penicillin, and 100 µg/mL streptomycin, all from Invitrogen). Cells were seeded into a cell‐repellent 96‐well plate (Greiner Bio‐One, Frickenhausen, Germany) in 300 μL SCM per well. Thereafter, the culture plate was placed on top of the Bioprinting Magnetic Plate (Greiner Bio‐One, Frickenhausen, Germany), which consists of a base with 96 neodymium magnets (0.0625″ OD, Nano3D Biosciences) designed to be located centrally in each well. Cells were incubated with the bioprinting system for 48 h in experiments I–IV and 72 h in experiments V‐VIII. The cell number per well and cell proportion (BOEC: BOSC) are described in each experiment. The variations on this protocol are described in the next sections.

#### 4.4.1 Spheroid Cell Number Size and BOEC: BOSC Proportion

In experiment I, different cell number sizes were evaluated. Independent of cell number size per spheroid, the media volume per well was always 300 μL. Therefore, different cell solutions were prepared according to the desired spheroid cell number size. For the spheroids that consisted of BOEC and BOSC, cell solutions were always prepared as 150 μL for BOEC and 150 μL for BOSC, totaling 300 μL per well. The specific calculations for cell number size and cell proportion are presented in Table 1.

**Table 1:** Specific cell solution concentration and volume seeded per well in different spheroid cell number sizes and BOEC: BOSC proportions.

Spheroid size	Proportion BOEC: BOSC	Concentration (cell/mL)		Volume per well (μL)	
		BOEC	BOSC	BOEC	BOSC
50,000	100%:0%	166,000	—	300	—
	70%:30%	116,000	50,000	150	150
25,000	100%:0%	83,000	—	300	—
	70%:30%	58,000	25,000	150	150
10,000	100%:0%	33,000	—	300	—
	70%:30%	23,000	10,000	150	150
	50%:50%	16,500	16,500	150	150
5000	100%:0%	16,000	—	300	—
	70%:30%	11,000	5000	150	150

#### 4.4.2 Magnetization by Overnight Incubation

In experiment II, the magnetization by overnight incubation was compared to the magnetization by centrifugation. For that, BOEC and BOSC under a monolayer culture (80% confluence) were incubated with NS (160 μL per T25 flask). Next, the cells were trypsinized, counted, resuspended in Spheroid Culture Media, and seeded in the well, as described before.

#### 4.4.3 Cell Seeding as One‐ or Two‐Step Protocol

The one‐step protocol was performed as described in Section 2.4, where both cell types (BOEC and BOSC) were trypsinized and seeded simultaneously into the well. For the two‐step protocol, the BOSC was processed on the first day and seeded into the well in 150 μL SCM per well, while the BOEC was processed 24 h later and seeded into the well in 150 μL SCM per well, totaling 300 μL. The incubation time with the Bioprinting Magnetic Plate was always timed after the seeding of the first cell.

#### 4.4.4 Extracellular Matrix Supplementation Into the SCM

As the ECM supplement, the Geltrex LDEV‐Free Reduced Growth Factor Basement Membrane Matrix (ThermoFisher, Cat. A1413201) was used in this study. Using a cold pipette tip, the Geltrex was added to cold SCM and homogenized. The 0.5–2% v/v Geltrex used in this study did not solidify once in the incubator.

### 4.5 Necrotic‐Positive Cell Ratio Evaluation (Propidium Iodide)

Propidium Iodide (PI) was used as a necrotic cell marker (Sigma, Cat. P4170), and Hoechst 33342 was used as a counterstain (ThermoFisher, Cat. H1399). Both dyes were added directly into the culture media and incubated for 3 h at a final concentration of 37.5 ng/mL per dye. Next, the spheroids were washed three times with warm PBS, fixed with 4% paraformaldehyde (PFA) for 15 min at room temperature, placed on glass slides with 1:1 glycerol: PBS, and immediately imaged. Z‐axis scans were acquired from each spheroid (10 μm per z‐stack) at 10× magnification under fluorescence microscopy (Leica Microsystems DM16000 B), and images were exported as Maximum Intensity Z‐projection. ImageJ‐Fiji (National Institutes of Health, Bethesda, MD, USA) was used to analyze the produced images. At least 200 cells were counted per spheroid, and the necrotic‐positive cell ratio was calculated as Ratio = (PI − T) * 100, where PI was the number of PI‐positive cells, T was the total cell number (PI + Hoechst) multiplied by 100, for percentage representation.

### 4.6 Spheroid Area and Spheroid Compaction Level Evaluation

The spheroid area and spheroid compaction level were measured by using the bright‐field (BF) pictures (Nikon microscope, 4× magnification). ImageJ‐Fiji (National Institutes of Health, Bethesda, MD, USA) was used to analyze the produced images. For that, images were first converted to 16‐bit, followed by an adjustment of the black‐and‐white threshold to distinguish cells from the free area (the same threshold was applied to conditions that would be compared). Using the freehand selection tool, a line was drawn on spheroids with dispersed cells, while the wand tool was used to compact spheroids. These regions of interest (ROI) were used to measure the area (presented as square millimeter value, mm^2^). The same ROI was applied to represent the compact spheroid level. For that, the proportion of the total spheroid area covered with dark pixels was used to represent the compactness level of the spheroids and presented as a percentage (%). This number qualitatively can be representative of the compactness level of spheroids, since the area corresponding to the dark pixels is representative of the cells' presence. Consequently, when there is a high dark pixel percentage, the cells are more attached, leaving fewer gaps (bright pixel area) in the spheroid area. The opposite is also true, a lower dark pixel percentage means more bright pixel areas, and so the cells are less attached, representing a less compact spheroid.

### 4.7 Immunofluorescent, Cytochemical Analysis, and Microscopy Analysis

For immunostaining, the spheroids were washed three times with warm PBS, and fixed in 4% PFA, w/v in PBS for 15 min at room temperature before storage in PBS at 4°C. The assay was performed as previously described (Ispada et al. 2020). Briefly, samples were permeabilized for 1 h in PBS with 0.5% Triton X‐100, followed by incubation in a blocking solution (2% bovine serum albumin in PBS with 0.5% Triton X‐100, 2 h, room temperature). Primary antibodies were incubated overnight at 4°C, with the dilution specified below. After washing (PBS with 0.5% Triton X‐100), the second antibodies were incubated for 2 h at room temperature (anti‐rabbit IGG Alexa Fluor 568, Cat. A10042, ThermoFisher, 1:300, and anti‐mouse IGG Alexa Fluor 488, Cat. Ab150109, Abcam, 1:300). After washing (PBS with 0.5% Triton X‐100), the spheroids were stained with Hoechst 33342 (15 μg/mL), and placed in glass slides with glycerol (1:1 in PBS), and immediately imaged. All the primary antibodies used in this experiment were reactive to bovine species: anti‐cytokeratin (Cat. C2562, dilution 1:1000), anti‐vimentin (Cat. C9080, dilution 1:500), anti‐acTUB (Cat. T6793, dilution 1:500), and anti‐OVGP1 (Cat. ab118590, Abcam, dilution 1:300).

Z‐axis scans were acquired from each spheroid (1 μm/z‐stack) at 20× and/or 40× magnification under confocal fluorescence microscopy (Leica SP8 Laser Confocal) or using fluorescence microscopy (Leica Microsystems DM16000 B). Images were analyzed by each z‐stack for OMS structure organization (cytokeratin and vimentin), or images were exported as Maximum Intensity Z‐projection for intensity analysis quantification (acTUB and OVGP1). ImageJ‐Fiji (National Institutes of Health, Bethesda, MD, USA) was used to quantify the total fluorescence intensities for acTUB and OVGP1, which were normalized by the spheroid area (measured as described in Section 4.6). Data is presented as the arbitrary unit of total fluorescence intensity by area.

For the native oviductal tissue staining, the oviducts were processed as described in Section 4.2. Next, a piece of the ampulla segment was snap‐frozen in liquid nitrogen before the cryosection procedure (8 μm transversal sections). Samples placed in the glass slide were fixed in ice‐cold acetone for 10 min at room temperature, followed by the permeabilization step as described above.

For the paraffin section samples, oviductal tissue (a piece of ampulla segment) and OMSs were fixed in 4% PFA, respectively during overnight and for 15 min incubation at room temperature. Samples were dehydrated in a series of alcohols and embedded in paraffin blocks. Transversal sections were obtained at 5 μm, which were dewaxed and rehydrated in a series of alcohols before staining with Hoechst 33342 (15 μg/mL, 30 min, room temperature), blue toluidine of 4.0 pH (for glycosaminoglycans and nucleic acids identified), and blue toluidine of 2.5 pH (for glycosaminoglycans identified only), following standard staining protocols (de Andrade Pinto et al. 2022). Finally, fluorescent samples were imaged using fluorescence microscopy (Leica Microsystems DM16000 B) at 20× magnification, and the cytochemical samples were imaged using an inverted light microscope (Zeiss, Axio Vert. A1) at 4× and 40× magnification.

### 4.8 Hormone Treatment in the OMS

P4 (Cat. P8783) and E2 (Cat. E8875) were diluted in absolute ethanol as a stock solution of 10 μg/mL and 0.1 μg/mL, respectively, storage at −20°C for no more than 1 month. For OMS treatment, Spheroid Culture Media was always freshly supplemented with hormone solutions. The ethanol final concentration in the culture media was no more than 1% v/v, which has been used in other studies (Chen et al. 2013a, 2018; Ferraz et al. 2018). The treatment protocol consisted of 7 days of luteal phase simulation (100 ng/mL P4), followed by 3 days of follicular phase simulation (300 pg/mL E2), and finally, 4 days of post‐ovulatory simulation (absence of hormone treatment), totaling 14 days of culture. The culture media (150 μL per well) were completely replaced every day. The hormone concentrations were designed based on the local bovine oviductal levels, which can be up to 50 times higher than the circulatory blood levels (Lamy et al. 2016). For the control group, only ethanol (0.8% v/v) was added to the Spheroid Culture Media, and media changing was performed similarly to the hormone‐treated groups.

### 4.9 Relative mRNA Abundance Quantification

The total RNA of individual OMS was extracted using the PureLink RNA Mini Kit (ThermoFisher), following the manufacturer's instructions, using 30 μL elution buffer in the final elution step. Each OMS was a replicate, and each experimental group had at least three OMS. Total RNA (10 μL/sample) was reverse‐transcribed using the High‐Capacity Kit (ThermoFisher). Quantitative PCR (qPCR) analysis was performed using the Taqman System (assays and master mix, ThermoFisher) in duplicate reactions. The qPCR amplification program consisted of denaturation at 95°C for 10 min, 40 cycles of 95°C for 20 s, and 60°C for 60 s. Relative quantification of RT‐qPCR data was calculated according to the 2^−ΔΔCT^ method (Livak and Schmittgen 2001). The actin beta gene (ACTB) was selected as the reference gene. All the Taqman probes were specific for *Bos taurus* species: ACTB (Bt03279174_g1), FOXJ1 (Bt04308989_m1) E2F1 (Bt04288961_g1), EGFR (AJT96D7), ESR1 (Bt03210039_m1), ESR2 (Bt03259200_m1), OVGP1 (Bt03253688_m1), and PGR (Bt07109254_m1).

### 4.10 Statistical Analysis

For statistical analysis, each spheroid was considered an experimental unit, represented as a circle in each graphical figure in the results. All statistical analysis was conducted using GraphPad Prism Version 8.4.3 software. Before statistical analysis, all obtained values were submitted to an outlier detection test (Grubbs' test with alpha = 0.05) and the normality test of D'Agostino and Pearson. Student *t*‐tests were performed for two‐group comparison and one‐way analysis of variance (ANOVA) with Tukey post hoc tests for comparison of three or more experimental groups. The statistical significance level was set at *p* < 0.05. All data are presented as mean ± standard deviation (SD) with single spheroid values represented in the graphs as a circle.

## Author Contributions

**Patricia Kubo Fontes:** conceptualization, methodology, formal analysis, validation, writing – original draft, writing – review and editing, investigation. **Ana Beatriz Florencio da Silva:** methodology, formal analysis, investigation, writing – original draft, writing – review and editing. **Ana Beatriz dos Reis Bartoli:** methodology, investigation, formal analysis, writing – original draft, writing – review and editing. **Thays Antunes:** methodology, investigation, formal analysis, writing – review and editing. **Arnaldo Rodrigues dos Santos Júnior:** methodology, formal analysis, writing – review and editing. **Marcella Pecora Milazzotto:** conceptualization, writing – review and editing, supervision, funding acquisition, project administration.

## Ethics Statement

All experimental procedures involving animals were approved by the Ethics Committee on the Use of Animals of the Federal University of ABC (UFABC, protocol 4682230223).

## Conflicts of Interest

The authors declare no conflicts of interest.

## Supporting information


**Supporting Figure 1 (PNG):** Frozen cell viability and cell population purity.

**Supporting Figure 2 (PNG):** Cell magnetization methods.

**Supporting Figure 3 (PNG):** Spheroid stability with culture media supplementation with extracellular matrix (ECM).

**Supporting Figure 4 (PNG):** BOEC‐ and BOSC‐only spheroids and BOEC/BOSC‐mix spheroids repeatability.

**Supporting Figure 5 (PNG):** Final Oviductal Magnetic Spheroid (OMS) culture protocol.

**Supporting Movie 1 (MP4):** Movie file of OMS stained with epithelial and stromal cell markers, related to Figure 3.

## Data Availability

Data generated or analyzed during this study are included in this published article and its supplementary information files. The data sets during and/or analyzed during the current study are available from the corresponding author upon reasonable request.
