# Supplementary figures and images for: Three‐Dimensional Magnetic Bioprinting Spheroids as an In Vitro Model to Study the Oviductal Physiology
Source: Mol Reprod Dev. 2025 Aug 21;92(8):e70049. doi: 10.1002/mrd.70049 (PMC12368830; doi:10.1002/mrd.70049)

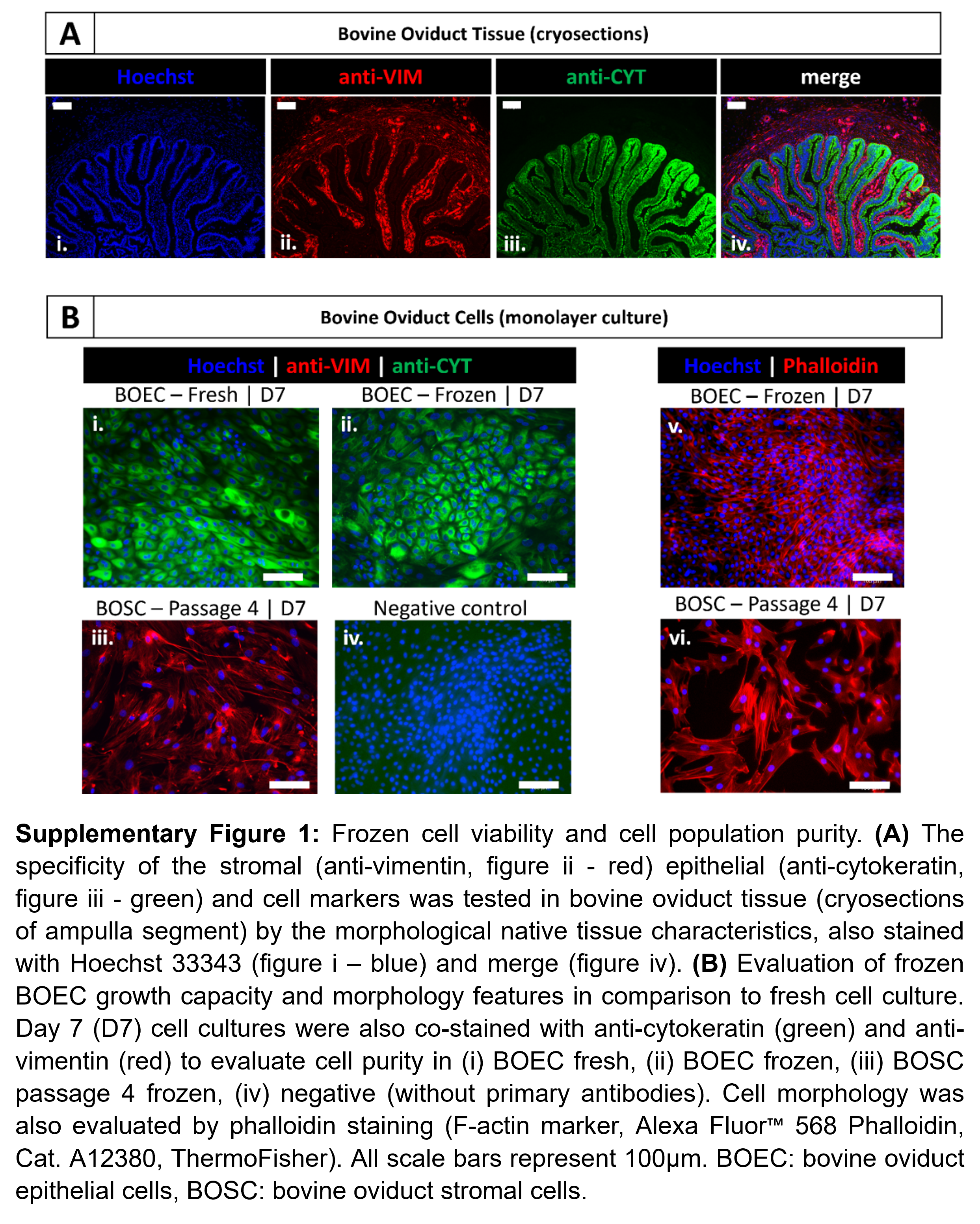

Supplement: Supplementary file 1 — Supporting Figure 1 (PNG): Frozen cell viability and cell population purity. [file MRD-92-e70049-s006.png]

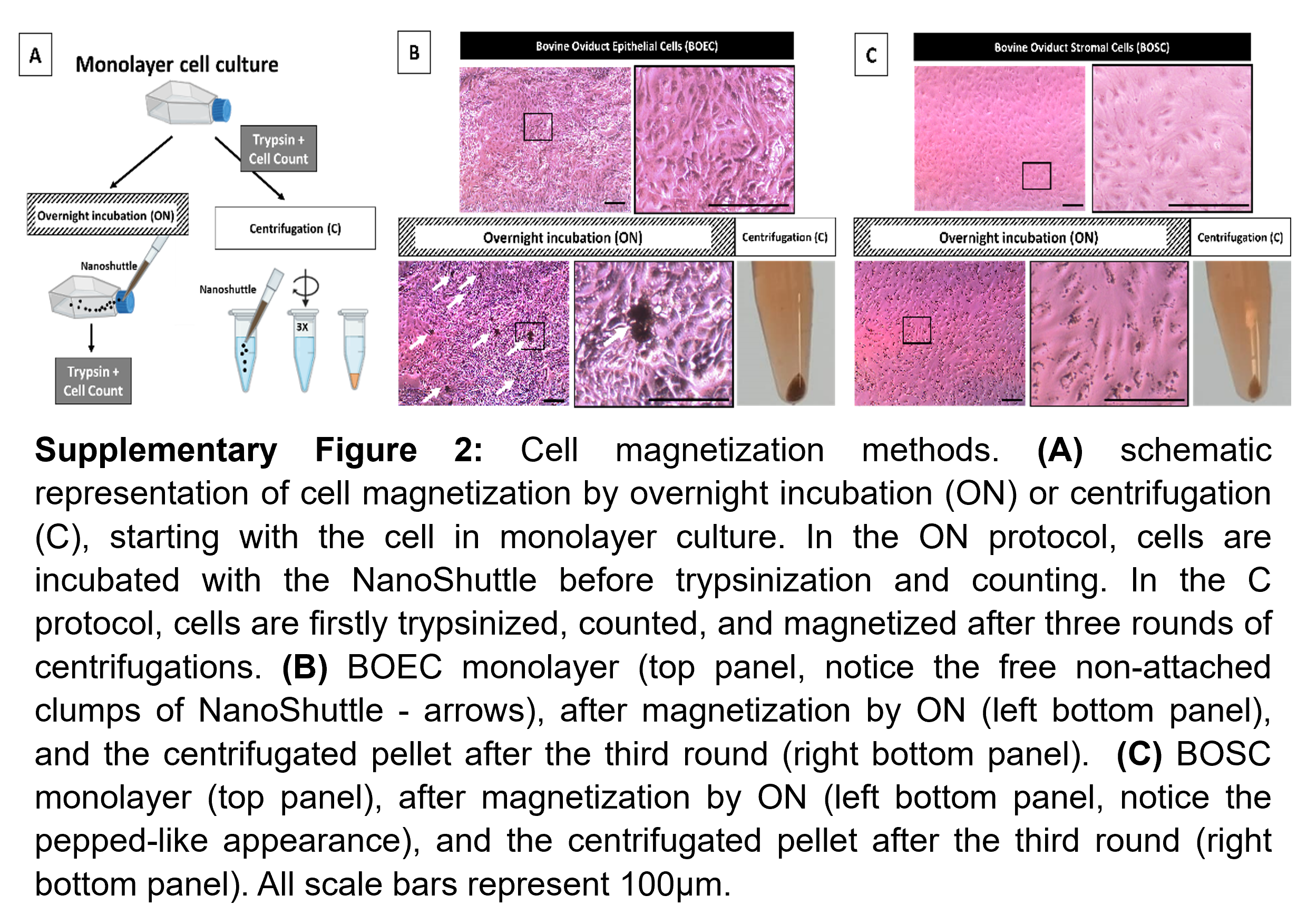

Supplement: Supplementary file 2 — Supporting Figure 2 (PNG): Cell magnetization methods. [file MRD-92-e70049-s005.png]

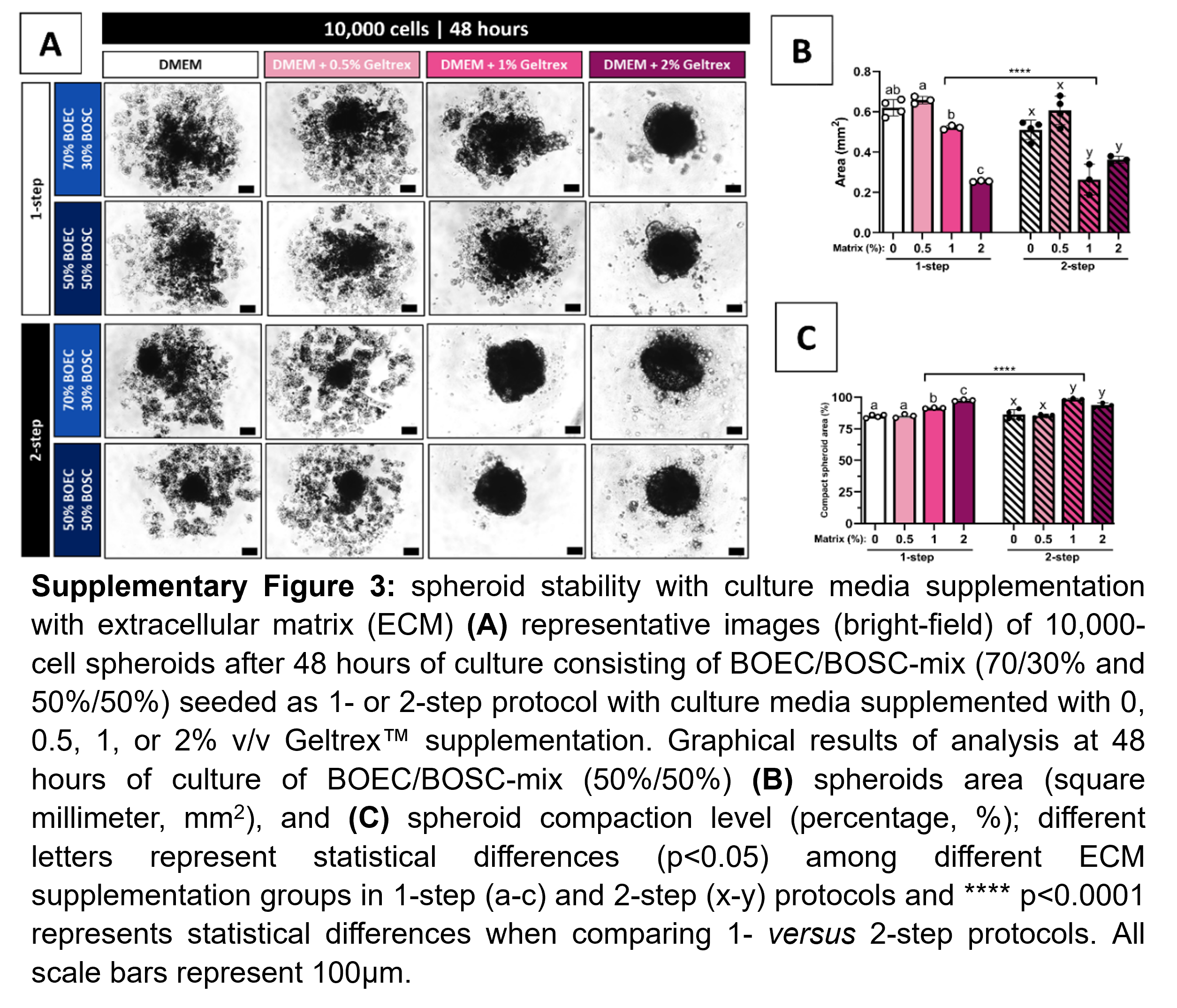

Supplement: Supplementary file 3 — Supporting Figure 3 (PNG): Spheroid stability with culture media supplementation with extracellular matrix (ECM). [file MRD-92-e70049-s004.png]

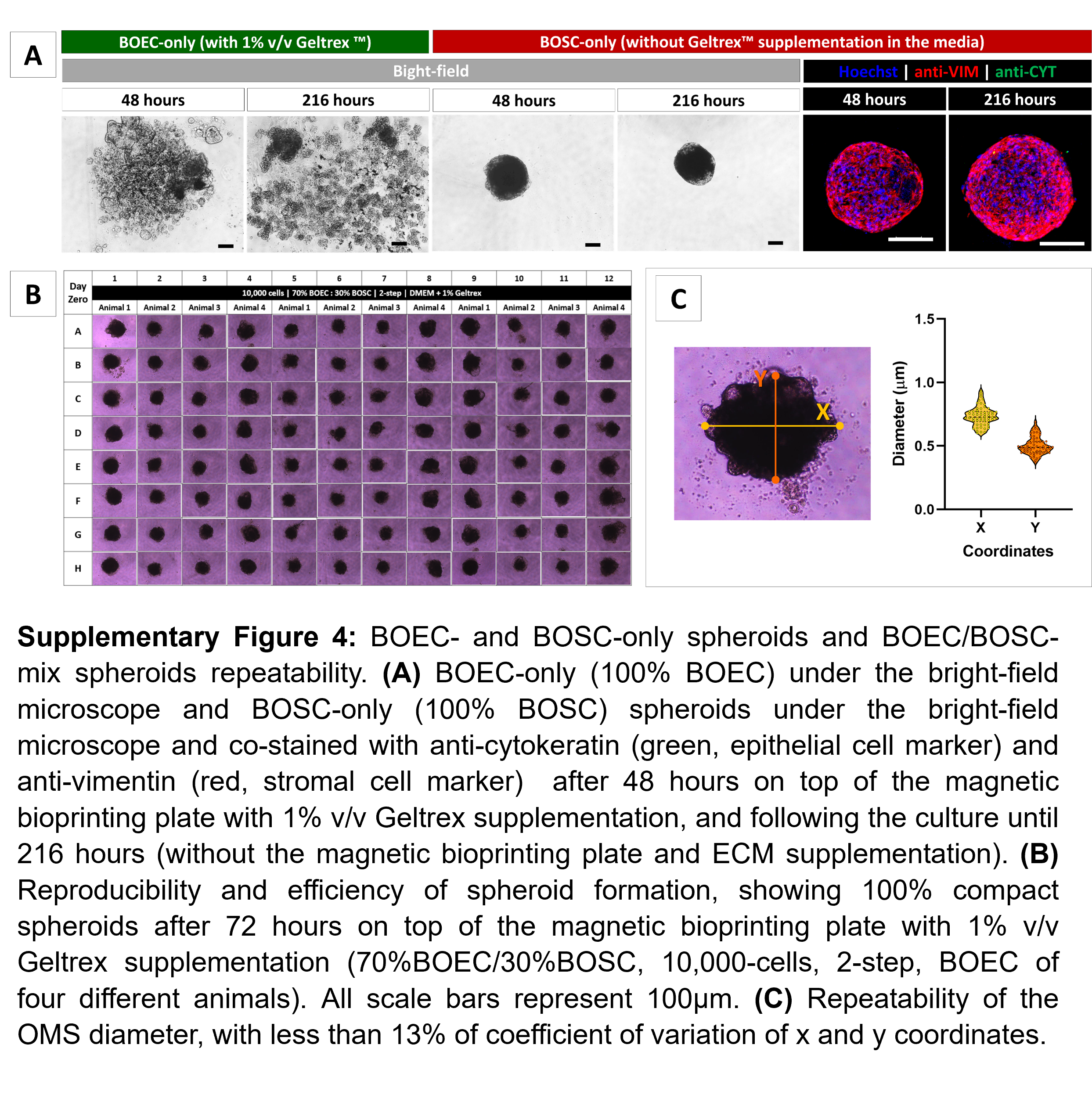

Supplement: Supplementary file 4 — Supporting Figure 4 (PNG): BOEC‐ and BOSC‐only spheroids and BOEC/BOSC‐mix spheroids repeatability. [file MRD-92-e70049-s002.png]

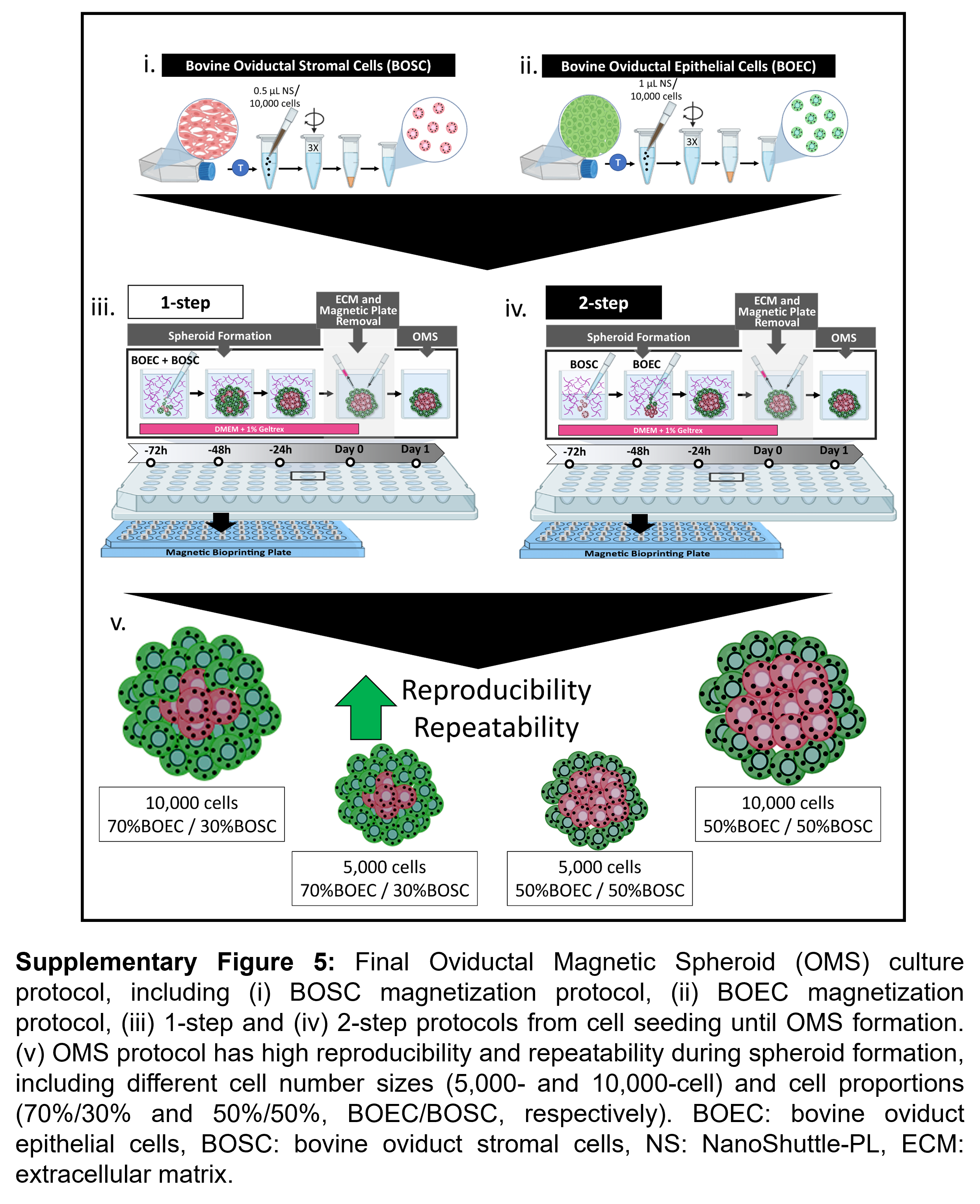

Supplement: Supplementary file 5 — Supporting Figure 5 (PNG): Final Oviductal Magnetic Spheroid (OMS) culture protocol. [file MRD-92-e70049-s001.png]
